# *insideOut*: A Bio-Inspired Machine Learning Approach to Estimating Posture in Robots Driven by Compliant Tendons

**DOI:** 10.3389/fnbot.2021.679122

**Published:** 2021-10-11

**Authors:** Daniel A. Hagen, Ali Marjaninejad, Gerald E. Loeb, Francisco J. Valero-Cuevas

**Affiliations:** ^1^Department of Biomedical Engineering, University of Southern California, Los Angeles, CA, United States; ^2^Ming Hsieh Department of Electrical and Computer Engineering (Systems), University of Southern California, Los Angeles, CA, United States; ^3^Division of Bio-Kinesiology and Physical Therapy, Departments of Computer Science and of Aerospace and Mechanical Engineering, University of Southern California, Los Angeles, CA, United States

**Keywords:** biologically-inspired robots, deep learning, artificial neural networks, tendon-driven systems, sensor fusion

## Abstract

Estimates of limb posture are critical for controlling robotic systems. This is generally accomplished with angle sensors at individual joints that simplify control but can complicate mechanical design and robustness. Limb posture should be derivable from each joint's actuator shaft angle but this is problematic for compliant tendon-driven systems where (*i*) motors are not placed at the joints and (*ii*) nonlinear tendon stiffness decouples the relationship between motor and joint angles. Here we propose a novel machine learning algorithm to accurately estimate joint posture during dynamic tasks by limited training of an artificial neural network (ANN) receiving motor angles *and* tendon tensions, analogous to biological muscle and tendon mechanoreceptors. Simulating an inverted pendulum—antagonistically-driven by motors and nonlinearly-elastic tendons—we compare how accurately ANNs estimate joint angles when trained with different sets of non-collocated sensory information generated via random motor-babbling. Cross-validating with new movements, we find that ANNs trained with motor angles *and* tendon tension data predict joint angles more accurately than ANNs trained without tendon tension. Furthermore, these results are robust to changes in network/mechanical hyper-parameters. We conclude that regardless of the tendon properties, actuator behavior, or movement demands, tendon tension information invariably improves joint angle estimates from non-collocated sensory signals.

## 1. Introduction

What are the control mechanisms by which Nature masters versatile limb movements that robots have yet to learn? Even the smallest of creatures can quickly and expertly learn to control their limbs for a variety of tasks, yet robots struggle to match this level of performance and generalizability. Therefore, roboticists often turn to Nature for inspiration, hoping to harness hidden lessons to build robots with comparable functional versatility. Tendon-driven robots are a bio-inspired class of robots that are becoming increasingly popular because of their functional advantages (Laurin-Kovitz et al., 1991; Lee and Tsai, 1991; Pratt and Williamson, 1995; Kobayashi et al., 1998; Pratt, 2002; Valero-Cuevas, 2016; Mazumdar et al., 2017; Marjaninejad and Valero-Cuevas, 2019; Marjaninejad et al., 2019a,b; Andrychowicz et al., 2020). Chief among these is the fact that tendon-driven robots are not burdened by the need to place actuators at the joints they actuate. They can instead be placed more proximally to provide more robust and efficient quadrupedal or anthropomorphic robots by reducing mechanical vulnerability and limb inertia (Jacobsen et al., 1986).

An estimate of the configuration of the limb is essential for most state-based robotic feedback control strategies (Heess et al., 2017; Marjaninejad et al., 2019b; Andrychowicz et al., 2020). In the absence of visual feedback, this is generally accomplished by placing sensors directly on joints. This increases limb inertia, complicates mechanical design, can be an additional source of noise, and risks damage (Marjaninejad et al., 2019b,c). These adverse effects become more pronounced for slender or deformable limbs (e.g., fingers in tendon-driven robotic hands) where it may be impossible or impractical to sensorize the joints. To address these challenges, we must ask, “*What alternatives are there to on-location joint sensors?*” and, more importantly, “*How has Nature evolved to solve these problems?*”

**Figure 1:**
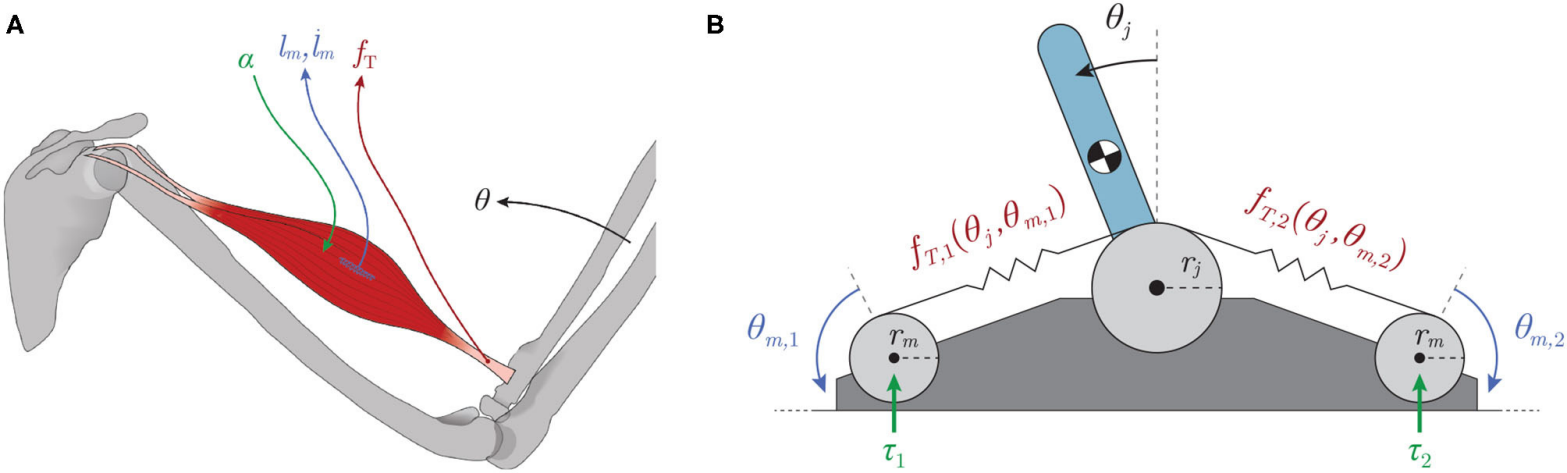
**(A)** Types of mechanoreceptors present in a muscle. Muscles are activated by spinal α-motorneurons (*green*) and any subsequent muscle fascicle (i.e., individual muscle fiber) length changes and velocities are sensed by the muscle spindles (*l*_*m*_ & ${\overset{∙}{l}}_{m}$, *blue*) while the Golgi tendon organs are responsible for detecting tendon tension (*f*_*T*_, *maroon*). **(B)** Schematic of a tendon-driven system with 1 kinematic DOF and 2 degrees of actuation (motors) that pull on tendons with nonlinear elasticity (creating a tension, *f*_*T,i*_). The motors were assumed to be backdrivable with torques (τ_*i*_) as inputs.

Interestingly, biological limbs do not have dedicated sensors to explicitly and uniquely encode joint angles. Instead, they have sensors for muscle (actuator) lengths and velocities (called *muscle spindles*, Figure 1A *blue*; Crowe and Matthews, 1964) and for tendon tensions (called *Golgi tendon organs*, Figure 1A *maroon*; Appenteng and Prochazka, 1984)^1^. Previous work established that, in general, a functional (yet indirect) relationship exists between the states that these proprioceptors measure and the kinematic states (like posture; Hagen and Valero-Cuevas, 2020), in support of the theory that these proprioceptive signals could, in principle, be integrated by the nervous system to form internal representations of posture (Scott and Loeb, 1994; Dimitriou and Edin, 2008; Van Soest and Rozendaal, 2008; Kistemaker et al., 2013). Where and how this sensory information is fused biologically to predict joint angles and/or limb posture is not understood.

While it is sometimes possible to derive analytical relationships among tendon tensions, motor rotations, and joint posture given the precise equations for its kinematics and dynamics, in practice it is often impractical or even impossible to obtain accurate and time-invariant analytical models of such nonlinear dynamical systems (Bongard et al., 2006; Marjaninejad et al., 2017). Furthermore, even if an accurate analytical model of the system were available, these relationships (*i*) would not generalize across changes in mechanical designs or tasks and (*ii*) will become increasingly inaccurate as the plant suffers mechanical changes due to either damage or normal wear and tear (Palli et al., 2012). Therefore, data-driven implicit models that can efficiently map between sensory information and estimates of states are preferred in practical applications (Bongard et al., 2006; Marjaninejad et al., 2018; Kwiatkowski and Lipson, 2019).

In Hagen et al. (2020), we estimated joint angles by training artificial neural networks (ANNs) with limited amounts of different sets of non-collocated sensory information collected from a simulated inverted pendulum driven by compliant tendons (Figure 1B) during random motor babbling. This preliminary work concluded that limited amounts of the so-called *Bio-Inspired Set* of sensory inputs (i.e., motor rotations/velocities *and* tendon tensions, Figure 1A) were sufficient to train ANNs to predict time-varying joint angles with reasonable accuracy (~2° average error). While these initial results were promising, the average error was perhaps too high to suggest that such a machine-learning approach to joint angle estimation could be used in real robots. Furthermore, no sensitivity analysis was performed to determine the robustness of such an algorithm to changes in the hyper-parameters of the neural networks (e.g., duration of motor babbling or number of hidden-layer nodes) or the mechanical parameters of the system.

Here we expand upon that framework to address these initial limitations and to fully develop an algorithm that accurately and reliably predicts joint posture from non-collocated sensors in a model tendon-driven robot (called *insideOut* to impress the importance of proprioception of *internal* variables to *outward* performance). We emphasize posture estimation via limited experience in robots because learning and operating in the physical world often precludes collecting large amounts of data, which is time-consuming and potentially damaging (Marjaninejad et al., 2019a). To that end, four main extensions were made to the original framework; (*i*) increasing the limit on the number of epochs that ANNs can use to refine their mapping when training with some set of babbling data (thus increasing overall performance); (*ii*) testing different types of motor babbling to more strategically explore the task space; (*iii*) exploring the trade off between the number of hidden-layer nodes and duration of motor babbling by testing whether and how changes to the number of hidden-layer nodes (previously fixed to 15) will affect the performance of these ANNs; and (*iv*) performing extensive sensitivity analysis across tasks and the mechanical parameters of the tendon-linked actuators (i.e., tendon stiffness and motor damping) to demonstrate its general utility for immediate application in physical robots.

## 2. Materials and Methods

### 2.1. Definition of the Plant and Its Dynamics

Our goal was to determine the utility of observing different kinds and amounts of sensory information in a tendon-driven system in simulation. It is impractical at this point to use a physical system that is prone to damage during the many trials required to develop and compare ANN methods. Future work will use physical systems in the now validated algorithm. We simulated a simple 1 degree of freedom (DOF) tendon-driven system with 2 actuators (the standard so-called *2N design*) that pull on tendons with nonlinear stiffness (Figure 1B). We modeled the motor actuators as idealized sources of torque (τ_*i*_) with no gearing to allow backdrivability. Similar to the approach taken in Palli et al. (2007), the tension on a tendon [*f*_*T,i*_, (1)] was modeled as an exponential function of tendon stretch (Δ*l*_*T,i*_) with positive scaling coefficient (*k*_*T*_) and rate constant (*b*_*T*_) to fit the shape and slope of this relationship. Tendon stretch was calculated as the difference between joint angle (θ_*j*_) and motor angle (θ_*m,i*_) excursions.


$$\begin{array}{ll} f_{T,i}(\theta_{m,i},\theta_{j})=\left\{ \begin{array}{l} k_{T}(\exp(b_{T}\Delta l_{T,i})-1);\;(\Delta l_{T,i}\geq 0)\\ 0;\;(\Delta l_{T,i}<0) \end{array}\right.\; & \begin{array}{l} \left(1\text{a}\right)\\ \left(1\text{b}\right) \end{array} \end{array}$$

$$\begin{array}{l} \text{where }\Delta l_{T,i}=\{\begin{array}{c} r_{m}\theta_{m,1}-r_{j}\theta_{j};\;i=1\\ r_{m}\theta_{m,2}+r_{j}\theta_{j};\;i=2 \end{array}\\ \text{and }b_{T}>0,k_{T}>0\text{ are shape constants.} \end{array}$$

$$\begin{array}{ll} \left\{ \begin{array}{l} \;{\ddot{\theta }}_{j}=\frac{1}{I_{j}}\left[-D_{j}{\dot{\theta }}_{j}-G(\theta_{j})+r_{j}(f_{T,1}(\theta_{j},\theta_{m,1})-f_{T,2}(\theta_{j},\theta_{m,2}))\right]\\ {\ddot{\theta }}_{m,i}=\frac{1}{I_{m,i}}\left[-D_{m}{\dot{\theta }}_{m,i}-r_{m}f_{T,i}(\theta_{j},\theta_{m,i})+\tau_{i}\right]\;(\text{for }i\in \{1,2\}) \end{array}\right. & \begin{array}{l} \left(2\text{a}\right)\\ \left(2\text{b}\right) \end{array} \end{array}$$

Therefore, the equations of motion for this system *without contact* are given by (2) where *G* is the torque due to gravity^2^, and *I*, *D*, and *r* represent the moment of inertia, damping coefficient, and moment arm for either the joint or the motors (denoted by the subscripts *j* and *m*, respectively)^3^. We can then rewrite the system of equations for *contactless* dynamics in its state space representation, (3)–(5), where $\vec{x}={\left[\theta_{j},{\overset{∙}{\theta }}_{j},\theta_{m,1},{\overset{∙}{\theta }}_{m,1},\theta_{m,2},{\overset{∙}{\theta }}_{m,2}\right]}^{T}$, $\vec{u}={\left[\tau_{1},\tau_{2}\right]}^{T}$, and $\vec{y}=h\left(\vec{x}\right)$ is the desired output (e.g., *y* = θ_*j*_). To approximate contact dynamics at the boundaries (±90° from vertical) without the use of high impedance boundary functions^4^, computational “hard stops” were invoked whenever the pendulum attempted to leave the range of motion by introducing a restoring joint torque that was equal to but opposite of any torque applied into the boundary (s.t. the right hand side of (2a) was zero when pushing on the boundaries at ±π/2).


$$\begin{array}{ll} \left\{ \begin{array}{l} \dot{\vec{x}}=f(\vec{x})+g(\vec{x})\vec{u}\\ \vec{y}=h(\vec{x}) \end{array}\right. & \begin{array}{l} \left(3\text{a}\right)\\ \left(3\text{b}\right) \end{array} \end{array}$$

(4) $$f(\vec{x})=\left(\begin{array}{c} x_{2}\\ Eq.(2\text{a})\\ x_{4}\\ Eq.(2\text{b}),\;i\;=1\\ x_{6}\\ Eq.(2\text{b}),\;i\;=2 \end{array}\right)$$

(5) $$g(\vec{x})=\left(\begin{array}{cc} 0 & 0\\ 0 & 0\\ 0 & 0\\ I_{m,1}^{-1} & 0\\ 0 & 0\\ 0 & I_{m,2}^{-1} \end{array}\right)$$

### 2.2. Choosing Tendon Stiffness Parameters

The goal of these experiments was to explore what types of sensory information are needed to accurately predict joint angles in a *compliant* tendon-driven robotic system. The parameters used to categorize *tendon stiffness* (i.e., *k*_*T*_ and *b*_*T*_) were initially chosen to simulate very compliant tendons so that we might understand if tendon tension information is vital to this estimation. We know from previous work that tendon compliance decouples the relationship between muscle and musculotendon *in vivo* and suspect that for a robotic system this decoupling makes it difficult to predict joint angles from motor angle and angular velocity measurements (Hagen and Valero-Cuevas, 2020). Therefore, we began by exploring a very compliant system as a worse case.


(6a) $$K_{j}=\frac{\partial }{\partial \theta_{j}}[r_{j}(f_{T,1}(\theta_{m,1},\theta_{j})-f_{T,2}(\theta_{m,2},\theta_{j}))]$$

(6b) $$\begin{array}{l} =k_{T}b_{T}r_{j}^{2}(\exp(b_{T}\Delta l_{T,1})+\exp(b_{T}\Delta l_{T,2}))\\ \;(\text{Assuming }\Delta l_{T,i}\geq 0\;for\;i\in \{1,2\}) \end{array}$$

(6c) $$=b_{T}r_{j}^{2}(f_{T,1}(\theta_{m,1},\theta_{j})+f_{T,2}(\theta_{m,2},\theta_{j})+2k_{T})$$

In order to choose (and subsequently vary) these parameter values for *tendon* stiffness, we imposed two constraints to ensure that the *joint* stiffness values [*K*_*j*_, (6)] fell within a conservative range ([10,50] Nm/rad) during a given movement, so long as the amount by which the tendon stretched stayed within a desired range. Specifically, it was assumed that the minimum joint stiffness was to be conserved across parameter choices (such that $K_{j}^{\min}=2k_{T}b_{T}r_{j}^{2}=10$ Nm/rad if Δ*l*_*T,i*_ = 0 for *i* ∈ {1, 2}) and that the largest induced joint stiffness would occur near the largest desired tendon stretch. To find an equation for this second constraint, we considered the special case where the pendulum was in equilibrium at the vertical position and at the maximum desired joint stiffness value. By the symmetry of the plant, the tendon tensions (and by definition, the stretching of the tendons) were maximal and equal such that $K_{j}^{\max}=2k_{T}b_{T}r_{j}^{2}\exp\left(b_{T}\Delta l_{T,i}^{\max}\right)=50$ Nm/rad.

**Figure 2:**
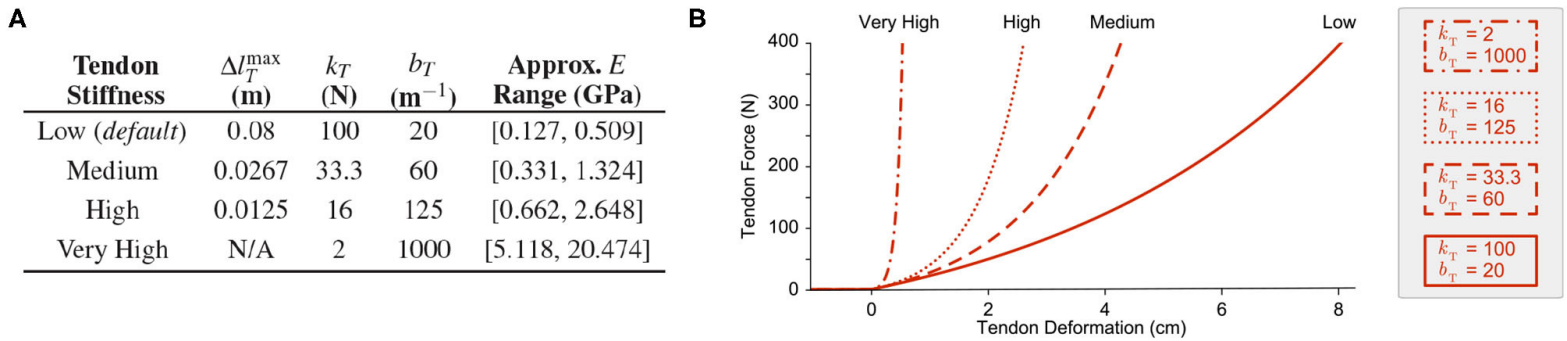
**(A)** Tendon stiffness parameters and the approximate range of Young's modulus values. **(B)** Examples of tendon tension-stretch curves for the parameters provided in **(A)**. The *Low* stiffness curve (*solid red*) represents the default force-length relationship for the compliant tendons proposed in Hagen et al. (2020).

From these two constraints it was possible to vary the parameters for tendon stiffness by changing the value for the largest desired tendon stretch. As in Hagen et al. (2020), we chose the maximum desired tendon stretch to be ~0.08 m for the default *Low* tendon stiffness, such that *k*_*T*_ = 100 N and *b*_*T*_ = 20 m^−1^. For the subsequent experiment where tendon stiffness was varied, we derived these parameters again using the aforementioned method for tendons that can stretch a maximum of 0.0267 and 0.0125 m (designated *Medium* and *High* tendon stiffness, respectively). But in the final experiment, where the tendons were made to be *nearly inextensible*, the parameters were chosen by observation and by satisfying the first constraint only (i.e., for increasingly stiff tendons, the maximum joint stiffness also increases). The parameter values used for these experiments are provided in Figure 2A and the respective tension-stretch relationships are plotted in Figure 2B.

To better comprehend the stiffness of these tendons, we compared the Young's modulus for each tendon when under ~400 N of tension (assuming that this tension corresponds to the same percentage of maximum stress, $\sigma_{T,i}^{\max}=f_{T,i}^{\max}/CSA$, and that each tendon had the same dimensions). Assuming a cylindrical tendon with a diameter of 0.001 m and a slack length (*l*_*T, s, i*_) between 0.01 and 0.04 m, the range for Young's modulus at this stress level (400N/*CSA*) was calculated by (8) (Figure 2A, right column).


(7) $$K_{T,i}=\frac{\partial f_{T,i}}{\partial (\Delta l_{T,i})}=k_{T}b_{T}\exp(b_{T}\Delta l_{T,i})$$

$$\begin{array}{ll} \begin{array}{l} E{|}_{f_{T,i}=400\text{ N}}=CSA^{-1}\cdot \left(K_{T,i}{|}_{f_{T,i}=400\text{ N}}\right)\cdot l_{T,s,i}\\ \;=CSA^{-1}\cdot b_{T}(400\text{ N}+k_{T})\cdot l_{T,s,i} \end{array} & \begin{array}{r} \left(8\text{a}\right)\\ \left(8\text{b}\right) \end{array} \end{array}$$

Note that the range of Young's moduli for the *Medium* and *High* stiffness tendons are 2.6x and 5.2x larger than the range for the default (rubberband-like) *Low* stiffness tendon, respectively. These higher stiffness values are more consistent with the observed range for physiological tendon (1.2–1.7 GPa), allowing us to speculate about just how useful physiological tendon sensors (i.e., Golgi tendon organs) are and their potential role in sensory fusion (Bennett et al., 1986; Zajac, 1989; Pollock and Shadwick, 1994)^5^.

### 2.3. Description of Improved Motor Babbling

Previously in Hagen et al. (2020), additive low frequency band-limited white noise was used to modulate the amplitude of random *ramp-and-hold* input signals in order to induce what is called “motor babbling.” As the average input levels of the motors during a given *hold* phase were uniformly sampled, it was rare for the inputs to be similar enough to each other for the limb to occupy the middle of the configuration space (even if only briefly). Instead, the effective “tug of war” happening between the tendons was typically dominated by one motor's random input level, causing the limb to rapidly flip across the configuration space to spend the remainder of the *hold* phase at that boundary. While this particular babbling strategy resulted in useful data after as little as 2 min (once the limb had flipped a sufficient number of times), it is not conducive to an efficient machine learning algorithm that relies on well-sampled data collected from *within* the configuration space and as such we aimed to develop a more strategic form of motor babbling to rectify this.

As these motors are effectively locked in a game of “tug of war” where the joint only moves if there is a *net* torque, the model spends more time in the mid-range of motion (collecting more useful data) if no motor is allowed to dominate by choosing input levels that “fight” to limit the net joint torque. To that end, we designed a motor babbling approach whereby motors are each assigned low frequency, band-limited white noise signals (1–10 Hz, 0–10 Nm) that (*i*) uniformly sample the input space and (*ii*) are made to be similar to each other (effectively emulating biological co-contraction of muscles). This more efficient form of motor babbling simultaneously explores the joint angle space whenever there is sufficient net torque for movement *as well as* its nullspace (i.e., the joint stiffness space) whenever the motors and tendons are “evenly matched.”

**Figure 3:**
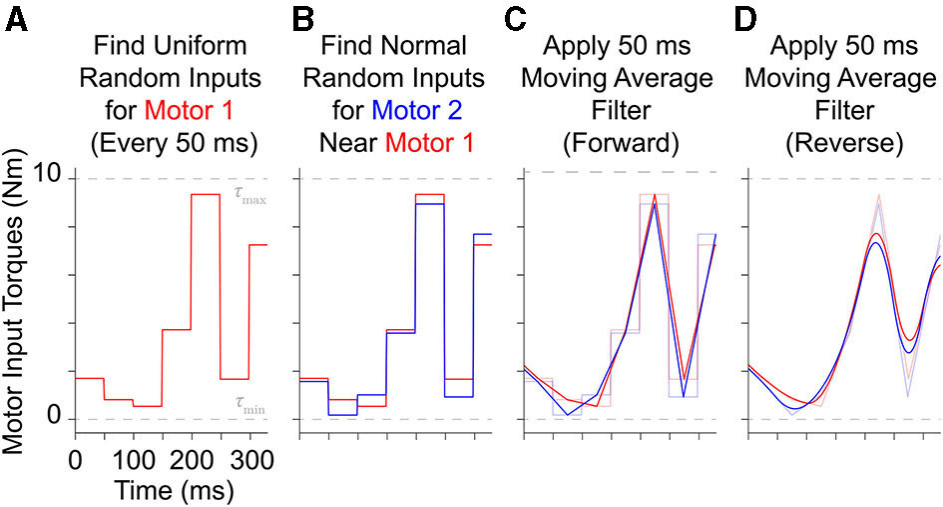
Example of how 300 ms of motor babbling signals are generated. **(A)** Random motor torque inputs are uniformly sampled from the range of possible inputs and assigned to 50 ms windows for motor 1 (*red*). **(B)** Then values for motor 2 (*blue*) are selected for each window from a normal distribution centered around the values for motor 1 with a standard deviation of 0.5 Nm (5% of the range of maximum input level). These discontinuous, piece-wise signals are then filtered using a forward **(C)** and backward **(D)** finite impulse response moving average filter with a filter lengths of 50 ms. This results in *correlated* band-limited, low frequency ( ≤ 10 Hz) white noise motor babbling signals.

To generate these motor babbling signals, the entire duration is first divided into 50 ms windows and then motor torque values are uniformly sampled and assigned to the first motor for each window (Figure 3A). Values for the second motor for each window are then randomly selected from *normal distributions centered around the first motor's random values* with a standard deviation of 0.5 Nm (Figure 3B)^6^. These discontinuous, piece-wise constant input signals are then filtered with a 50 ms moving average, finite-impulse response filter both forward and backwards to produce random motor babbling signals that are smooth, low frequency ( ≤ 10 Hz), and slightly correlated with each other (Figures 3C,D). These motor babbling inputs are then used to drive the plant in a feedforward simulation where the resulting joint angles are recorded along with all motor data (angles, angular velocities, and angular accelerations) and the tendon tension data (including their first and second derivatives). By choosing inputs that are often (nearly) equal, the limb stiffens during the resulting tendon “tug of war.” This new approach effectively samples the configuration space *near* the joint torque nullspace whenever the input levels are consistent with movement, allowing the limb to flip less rapidly across the configuration and for more useful data to be gathered from the joint's mid-range of motion. This is not unlike the co-contracted movements generated by infants during motor babbling (Dominici et al., 2011; Marjaninejad et al., 2019b).

### 2.4. Training and Testing Artificial Neural Networks

Similar to Hagen et al. (2020), for each babbling trial, four different sets of sensory information were generated using combinations of motor and/or tendon tension data and used to train ANNs to predict joint angles; the set of *All Available Data* (9), the *Bio-Inspired Set* (10), the set of *Motor Positions and Velocities Only* (11), and the set of *All Motor Data* (12).



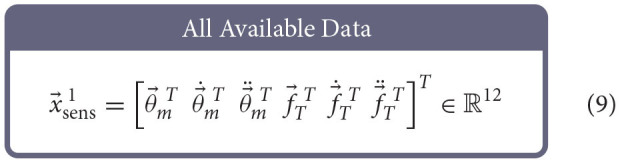





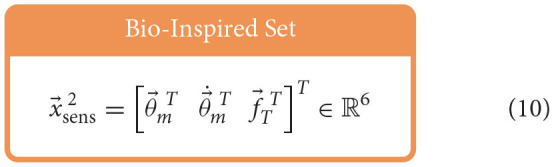





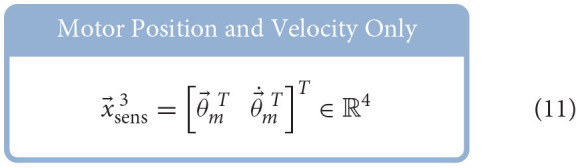





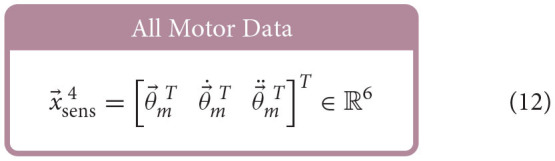



Feedforward neural networks were generated using MATLAB's Deep Learning toolbox, which allows for direct control over (*i*) the number of nodes in our hidden-layer, (*ii*) the way in which the weights and biases of the activation functions were initialized, and (*iii*) the type of optimizer used^7^ For each ANN created, the number of input layer nodes was equal to the number of elements in the sensory set and the number of output layer nodes was always one. Each network had one hidden-layer and the number of hidden-layer nodes was explored to determine the effect that it had on the performance of each network.

The function used to initialize the weights and biases of the network was changed from the previously-used, default function (Nguyen-Widrow initialization algorithm^8^) to one that ensured the weights and biases were initialized randomly each trial so that a proper comparison could be made between networks *on average*. The optimizer was chosen to be the Levenberg-Marquardt algorithm (a nonlinear least squares optimization technique) as it is very robust for minimizing mean squared error (MSE). As we wished to compare how well these ANNs trained with the four sensory sets were able to predict the joint angle for a simple 1 DOF the system, we will use the mean absolute error (MAE) to discuss performance, but for systems with higher DOFs mean squared error (or *root* mean squared error, RMSE) would be more appropriate. Therefore, this choice of optimizer was appropriate to find the (locally) optimal performance for these ANNs.

**Figure 4:**
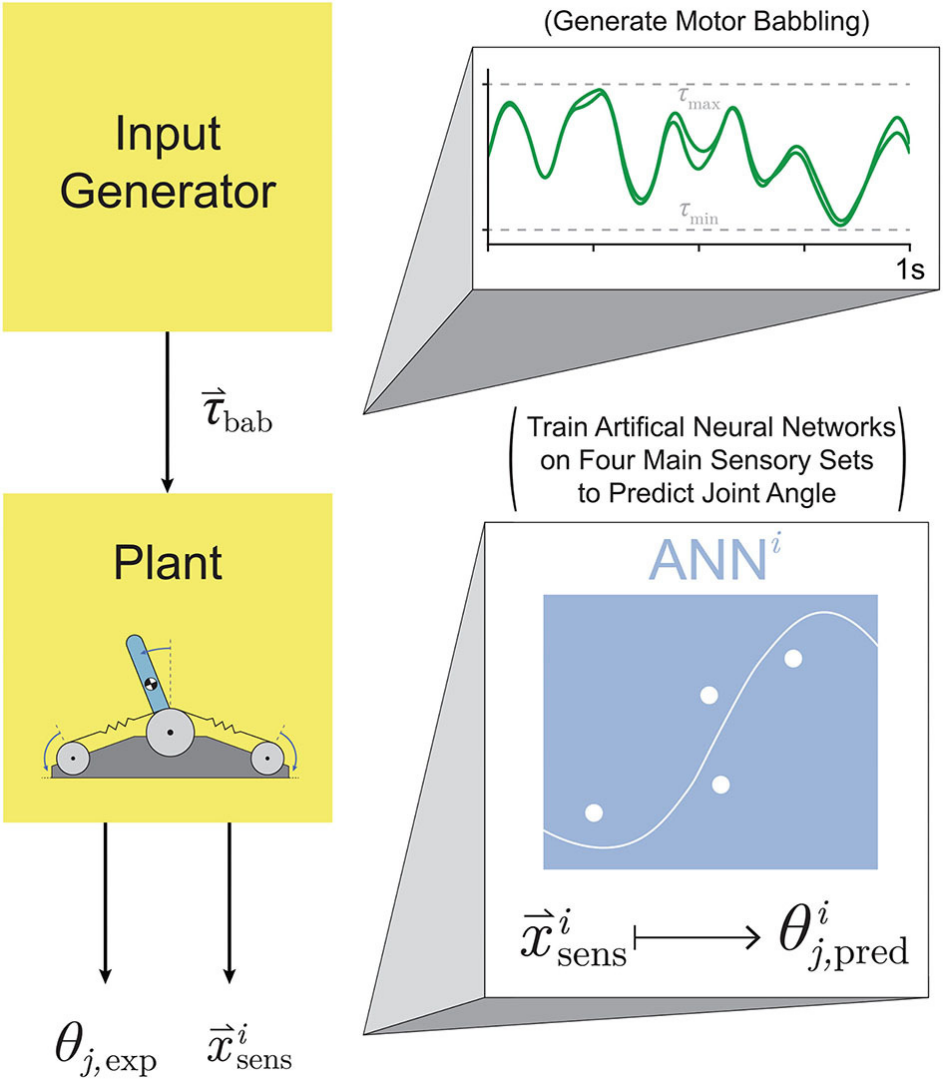
Proposed setup for training ANNs on motor babbling. Random input torques were generated from low frequency, band-limited white noise (1-10 Hz) chosen such that the difference between the two signals forms a normal distribution (0±0.5 Nm). These motor babbling signals (τ_bab_) are passed through the plant, the subsequent sensory information is recorded, and ANNs are trained with the four main sensory sets (${\vec{x}}_{\text{sens}}^{i}$) to predict joint angle ($\theta_{j,\text{pred}}^{i}$).

The general framework for generating and training these ANNs is illustrated in Figure 4. For each babbling trial and each sensory set, the babbling data was randomly divided into three sets; *training* (70%), *validation* (15%), and *testing* (15%). Validation checks were performed after each epoch and terminated the training if (*i*) the performance trended *worse* for 6 consecutive epochs or (*ii*) if the gradient of the performance was below a certain threshold (10^−7^, i.e., if the performance curve had flattened out and was near a minimum). The number of epochs that the networks can train on can also be modified to prevent overfitting (as well as to reduce training time), but because we were interested in the best performance possible by ANNs trained with these sensory groups, the epoch limit was changed from 50 to 10,000 to allow (most) networks to converge by means of the aforementioned validation checks instead.

### 2.5. Defining Different Movements to Test Generalizability

To adequately address each ANNs ability to generalize, we needed to identify movement tasks that were representative of most typical movements. An enabling feature of *nonlinearly-compliant* tendon-driven systems is the ability to control joint angle independently of joint stiffness [*K*_*j*_, (6)] by choosing different tendon tensions in the nullspace of the joint dynamics. Therefore, by defining four different movement tasks where joint angle and stiffness were prescribed either sinusoidal or random point-to-point trajectories within the range of these values, we were able to generate movement tasks that categorized most typical movements.

The sensory sets associated with each of these movements were generated using a feedback linearization algorithm [defined in Palli et al., 2008, and described in Hagen et al. (2020)]. Previously, point-to-point generalization trajectories were generated from piece-wise continuous reference trajectories that were only twice differentiable. As the feedback linearization algorithm utilized up to the fourth derivative of joint angle and up to the second derivative of joint stiffness *eq*. (6) for the control policy, the point-to-point reference trajectories needed to be changed in order to prevent large transients from occurring in the control. Even if only briefly, these transients can cause large errors in joint angle estimation across ANNs whenever the sensory input data are outside of the bounds learned during motor babbling which are used to normalize the inputs to the ANNs. We therefore created a point-to-point transition trajectory that would arrive and leave a given point with zero velocity, acceleration, jerk, and snap so that transitions between *ramp* and *hold* phases were continuously differentiable up to the fourth derivative (13).


(13) $$\begin{array}{l} y=y_{i}+\left(y_{f}-y_{i}\right)126\tau^{5}-420\tau^{6}+540\tau^{7}\\ \;-315\tau^{8}+70\tau^{9} \end{array}$$

where τ = (*t* − *t*_*i*_)/(*t*_*f*_ − *t*_*i*_) and *y* ∈ {θ_*j*_, *K*_*j*_}. By limiting the amount of time it takes to transition between points (*t*_*f*_ − *t*_*i*_) to a conservative (2 · 2Hz)^−1^ = 0.25 s we limited the frequency content of the point-to-point reference trajectories to be below 2 Hz. The output variable could then be held constant for a given duration (minus the transition time), before transitioning to another point.

**Figure 5:**
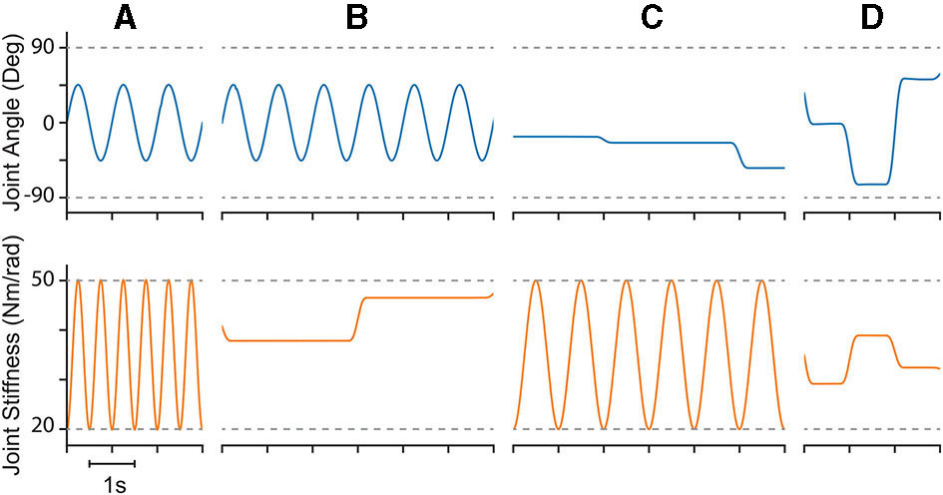
Example plots of four different types of reference trajectories used to test generalizability; **(A)** sinusoidal joint angle and sinusoidal joint stiffness, **(B)** sinusoidal joint angle and point-to-point joint stiffness, **(C)** point-to-point joint angle and sinusoidal joint stiffness, and **(D)** point-to-point joint angle and point-to-point joint stiffness. For point-to-point tasks, transitions were limited to 0.25 s (2 Hz cutoff) and designed in a way that they were continuously differentiable up to the fourth derivative (i.e., they left and arrived at each point with zero velocities, accelerations, jerks, and snaps).



Therefore, we defined the four movement trajectories used to test generalization trajectories as follows. When both joint angle and joint stiffness were made to follow sinusoidal trajectories, the joint angle was prescribed a sinusoidal trajectory of ±45° from vertical with a frequency of 1 Hz, while the joint stiffness was prescribed a cosine trajectory with twice that frequency such that maximum stiffness occurred at the extremes of the movement [or minimum stiffness occurred when swinging across the configuration space, (14), Figure 5A]. For this and all other trajectories, the range of joint stiffness values was chosen to be [20,50] Nm/rad. The length of this reference trajectory was chosen to be 10 s (10 periods of joint rotations), but once the controller converged on this periodic trajectory, so too did the sensory data associated with it. Therefore, because the sensory information will be periodic, the performance of each ANN will be periodic as well and only one period is needed to capture the performance behavior. We used the last three periods to ensure capture of the average behavior.

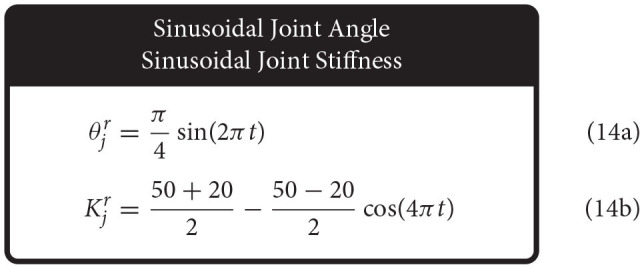



When the joint angle was made to follow a sinusoidal trajectory but the joint stiffness was made to follow a point-to-point task, the joint angle trajectory was prescribed as described above (1 Hz oscillation, ±45° from vertical), and the joint stiffness point-to-point task was chosen such that (*i*) the point-to-point values uniformly sampled the stiffness range and (*ii*) the step duration was equal to 3 times the period of the joint angle trajectory (Figure 5B). This ensured that the maximum positive angular velocity had two periods where it did not coincide with the transition to another stiffness point. Therefore, for each new random joint stiffness value, (1) was used to generate a smooth transition from the previous value with the appropriate boundary conditions, where it was held constant for 2.75 s before transitioning to the next value [step duration minus the transition time, (15)]. To adequately sample the joint stiffness space, we completed 100 point-to-point steps for this trajectory (300 s total).



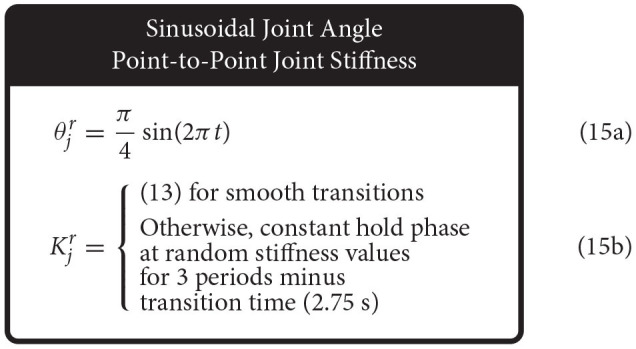



Conversely, when the joint stiffness was made to follow a sinusoidal reference trajectory and the joint angle was made to perform point-to-point tasks, a 1 Hz cosine trajectory was prescribed for the joint stiffness (spanning the joint stiffness range) while the joint angle was made to follow a random point-to-point task that uniformly sampled the *entire* range of motion with a step duration 3 times the period of the joint stiffness trajectory (16) (Figure 5C). As before, to adequately sample the joint angle space, we completed 100 point-to-point tasks for this trajectory (300 s).



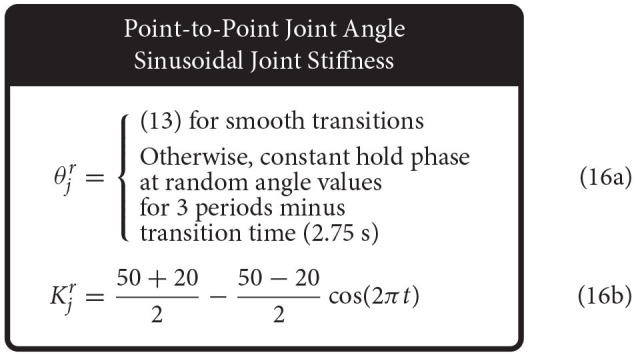



Lastly, when both joint angle and joint stiffness were made to follow point-to-point trajectories, the random values were uniformly selected from the full range of motion and the joint stiffness range, respectively (Figure 5D). The step duration was set to 1 s (minus the transition time), to ensure that the plant had ample time to converge to each point in joint angle and joint stiffness space. For this trajectory, 200 separate point-to-point tasks were completed (200 s total).



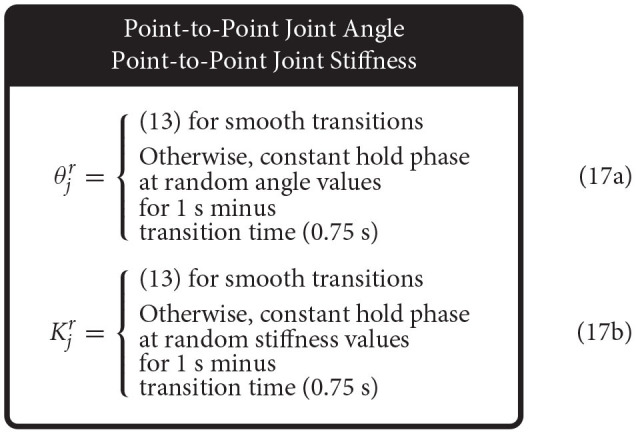



### 2.6. Sweeping Babbling Duration and Hidden-Layer Nodes

**Figure 6:**
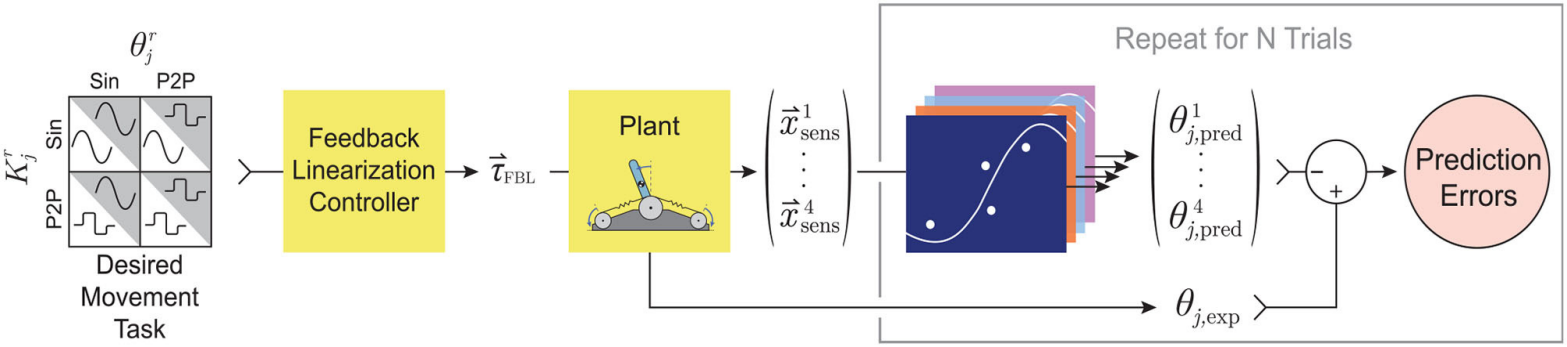
Proposed experimental setup for a *single* choice of either babbling duration (Experiment 1, *N* = 25) or the number of hidden-layer nodes (Experiment 2, *N* = 10). For either experiment, for each choice of the independent parameter, *N* motor babbling experiments were conducted and *N* ANNs were trained (see Figure 4). The performance of each of these networks was determined by its ability to generalize to different movements [where joint angle and/or stiffness are prescribed either sinusoidal (Sin) or point-to-point (P2P) trajectories; See Figure 5]. A feedback linearization controller then calculated the input torques needed to produce the desired movements (see Palli et al., 2008 or Hagen et al., 2020), which were then passed through the plant to produce the experimental joint angle (θ_*j*, exp_) as well as the four sensory sets of interest (${\vec{x}}_{\text{sens}}^{i}$). These sets were then passed through their corresponding ANNs (that were trained with babbling data) to predict joint angle ($\theta_{j,\text{pred}}^{i}$). The prediction errors for each network were then averaged over all trials, and the performance as a function of the independent parameter could then be evaluated.

With the general framework defined for training an ANN on a given sensory set, we considered how (*i*) motor babbling duration and (*ii*) the number of hidden-layer nodes affected the performance of the various ANNs. To test these, two experiments were conducted in which these parameters were varied while the *average* performance was determined for each sensory set across the four generalization movements. For either experiment, a total of *N* ANNs were trained for each sensory set from *N* babbling trials for each choice of the varied parameter, where *N* = 25 when motor babbling duration was varied and *N* = 10 when the number of hidden-layer nodes was varied. These ANNs were then asked to predict joint angles for the four generalization movements and the MAE was measured (Figure 6). The average MAE was then calculated for each sensory set to measure how well *on average* networks of these types could generalize given the choice of the varied parameter.

For the first experiment where we varied the babbling duration, we selected values between 1 and 25 s ({1} ∪ {2.5, 5, …, 25}) while initially assuming ANNs to have 15 hidden-layer nodes as previous work had shown that to be sufficient when *controlling* the joint angles of a tendon-driven system with *inextensible tendons* (Marjaninejad et al., 2019a). The results from this experiment (discussed in more detail below) showed that (*i*) average performance of all sets/movements and (*ii*) their relative standard deviations converged after 10–15 s. From these observations, the duration of motor babbling was fixed to 15 s for the second experiment where the number of hidden-layer nodes was varied from 1 to 19 with a resolution of 2 nodes.

### 2.7. Sweeping Movement Frequency

To observe how well ANNs trained with these sensory sets can generalize to joint angle sinusoidal movements of varying frequencies, we defined 8 separate movements where the angular frequency was chosen to be either 0.5, 1, 2, or 4 Hz and the joint stiffness was either varied sinusoidally or as a point-to-point task [(18)–(19) for *f* ∈ {0.5, 1, 2, 4} Hz].



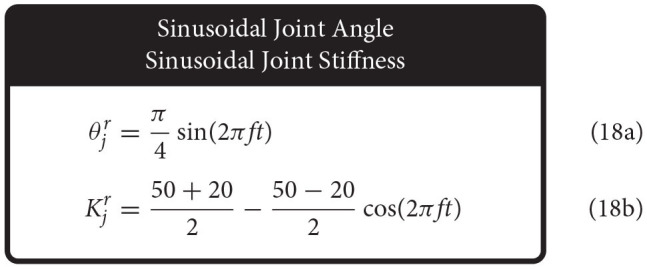





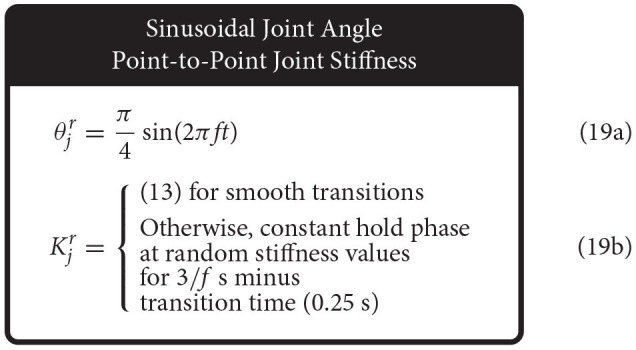



For each of these movements, the feedback linearization algorithm derived in Palli et al. (2008) was again used to generate the associated sensory sets. Assuming 15 s of motor babbling and 15 hidden-layer nodes, 50 ANNs were created for each sensory set from 50 trials of motor babbling which were then asked to predict the joint angles for each of these 8 generalization movements. As before, each ANN's performance was calculated as the MAE and the overall generalizability of each sensory set (for each movement) was calculated as the average across all 50 trials.

### 2.8. Sweeping Tendon Stiffness and Motor Damping

The next experiment swept across both tendon stiffness and motor damping parameters to better understand the affect those parameters have on performance. The approach used for each choice of parameter values was similar to the approach taken previously—ANNs for each sensory set were trained with motor babbling data and their overall (average) performance was determined by their ability to generalize to different movement tasks. As the control needed to produce the generalization trajectories changes whenever the plant is changed, the sensory sets associated with the four generalization trajectories must be generated via the feedback linearization algorithm for each of the 9 parameter settings described below (3 motor damping and 3 tendon stiffness). Then for each parameter setting, 50 ANNs were trained for each sensory set from 50 motor babbling trials to predict joint angle (assuming 15 hidden-layer nodes and 15 s of motor babbling) which were then asked to predict joint angles for each of the four generalization movements *uniquely generated for each parameter setting*. Consistent with previous experiments, each ANN's performance was calculated as the MAE and the overall generalizability of each sensory set (for each movement) was calculated as the average across all 50 trials. Therefore, for each of the 9 parameter settings chosen, a single performance metric was provided for each sensory set and each movement to describe how well each ANN generalized, allowing us to identify any trends across either parameter.

As previously discussed, the original choice of tendon stiffness was quite low in order to determine if tendon tension information would be useful for predicting joint angles in a very compliant tendon-driven system. However, to address whether this extreme choice of compliance affects the results, we utilized *Medium* and *High* stiffness tendons (defined previously) to compare the results. Additionally, the motor damping values of interest were chosen to be 0.5x, 1x, and 2x the nominal value provided in Palli et al. (2008) (0.00462 Ns/m).

### 2.9. Very High Tendon Stiffness Experiment

We were limited in how stiff we could make the tendons while still maintaining these joint angle reference trajectories with a joint stiffness range of [20,50] Nm/rad. At some point, the tendon stiffness became high enough that the range of possible tendon tensions needed to follow the prescribed joint angles would not intersect with the range of tendon tensions that produced these lower joint stiffness values (i.e., the movement became infeasible). Therefore, for a separate experiment where parameters *k*_*T*_ and *b*_*T*_ were chosen to describe a tendon with *Very High* stiffness (one that deformed nearly a tenth as much as the default *Low* stiffness tendon, bottom row in Figure 2A) new generalization trajectories were generated from a more reasonable joint stiffness range. This allowed us to answer the question, “What happens when tendon stiffness approaches infinity and the tendons become inextensible?”

While conducting a preliminary motor babbling experiment with these new tendon stiffness parameters, it was observed that the range of induced joint stiffness values was quite large with a maximum of around 2,000 Nm/rad. Therefore, it was determined that keeping the joint stiffness between 150 and 650 Nm/rad for the four generalization trajectories would (*i*) better represent the typical joint stiffness values this plant would experience during babbling while (*ii*) allowing the feedback linearization algorithm to adequately control these trajectories.

As we did for the previous experiments, generalization trajectories were generated using the feedback linearization controller and the resulting sensory information was divided into the four sensory sets. Then 50 ANNs were generated for each sensory set (15 hidden-layer nodes) and trained with random babbling data (15 s) to predict joint angle. The ability of each ANN to generalize was measured as the MAE for each of the four movement trajectories and the average performance was then calculated to identify how these performances compared to the considerably lower stiffness examples seen in previous experiments.

## 3. Results

### 3.1. Sweeping Motor Babbling Duration

**Figure 7:**
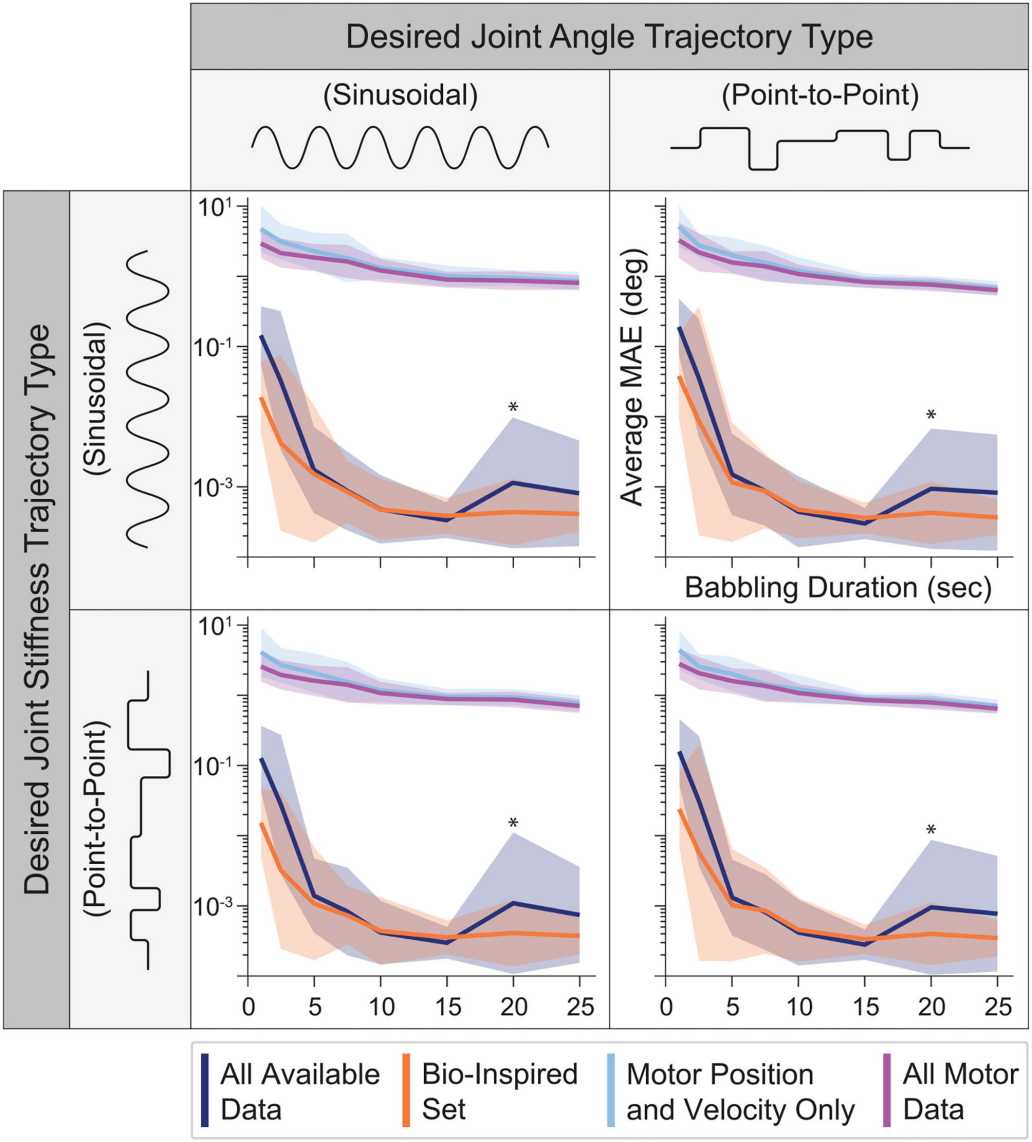
The average performance (MAE) and relative standard deviation (*shaded region*) for each sensory set (*N* = 25 ANNs, 15 hidden-layer nodes) plotted against the babbling durations used to train these networks for all four generalization movements. Those ANNs trained with tendon tension (*dark blue* and *orange*) drastically outperform those trained only with motor information (*light blue* and *purple*) with a 3 orders of magnitude improvement after as little as 5 s of training. Additionally, the *Bio-Inspired Set* performed as well as our baseline set (*All Available Data*), suggesting that tendon tension in addition to motor positions and velocities are sufficient to predict joint angles. *Note that because this plotted on a log scale, the peaks for both the *All Available Data* and *Bio-Inspired Set* at 20 s of babbling are on the order of 10^−3^ to 10^−2^, and therefore do not reflect large variations from the average values but more likely noise.

When motor babbling duration was varied between 1 and 25 s (and the number of hidden-layer nodes was fixed to 15), we found that ANNs trained with tendon tension data (i.e., the set of *All Available Data* and the *Bio-Inspired Set*), provided more accurate joint angle estimates across all babbling durations and generalization movements (Figure 7). In fact, ANNs that utilized tendon tension information performed nearly 2–3 orders of magnitude better with as little as 5 s of motor babbling. Interestingly, ANNs trained with the *Bio-Inspired Set* performed as well as (if not better than) those trained with the set of *All Available Data*. Additionally, we can see from Figure 7 that both the performance *and* relative standard deviation converge for all sensory sets after as little as 10–15 s of motor babbling^9^. Therefore, we adopted a reasonable 15 s of motor babbling when we explored changes in performance as a function of nodes in the hidden layer.

### 3.2. Sweeping Number of Hidden-Layer Nodes

**Figure 8:**
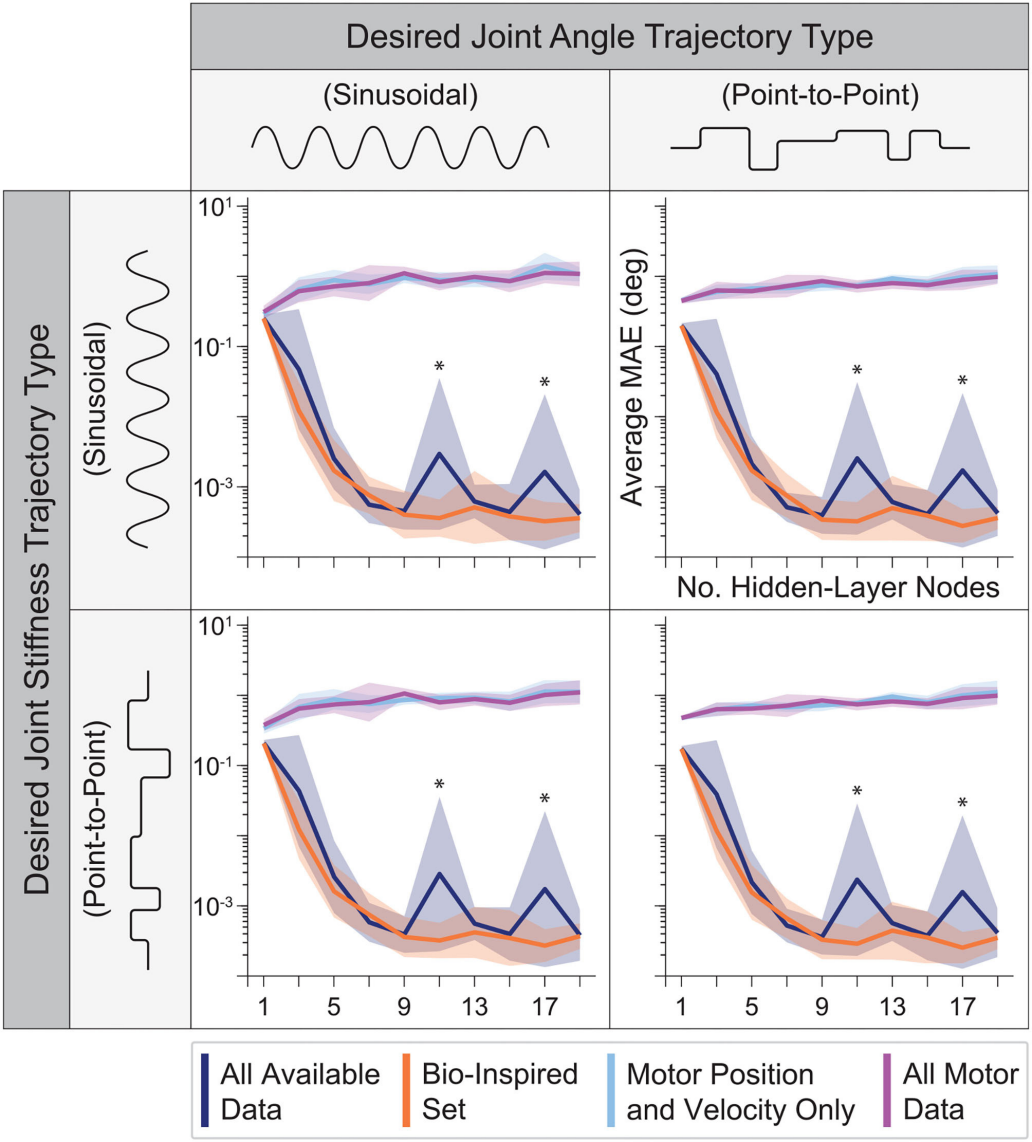
The average performance (MAE) and relative standard deviation (*shaded region*) for each sensory set (*N* = 10 ANNs, 15 s of motor babbling) plotted against the number of hidden-layer nodes used by each ANN for all four generalization movements. For any choice in the number of hidden-layer nodes, the ANNs with tendon tension outperformed those trained only with motor information. However, for fewer and fewer hidden-layer nodes, the performance of the ANNs trained without tendon tension improved while the performance of ANNs trained with tendon tension data degraded *but* always performed best. It can be seen that the performance of the ANNs trained with tendon tension data begin to plateau for 9+ hidden-layer nodes. *Note that because this plotted on a log scale, the peaks for *All Available Data* at 11 and 17 nodes are on the order of 10^−3^ to 10^−2^, and therefore do not reflect large variations from the average values but more likely noise.

The performance of ANNs trained with tendon tension information increases rapidly across all movements and then saturates as the number of nodes increases (Figure 8). Intuitively, if the number of nodes is too small, features of the data will be discarded as there are fewer available ANN parameters to capture it and the performance degrades. It is important, however, to note that their performance was never worse than that of the ANNs trained without tendon tension. Interestingly, their performance saturated after 9 hidden-layer nodes (even though using as little as 3 nodes still resulted in approximation errors less than 10^−2^ degrees on average). Thus, our original choice of 15 nodes for the ANNs in the first experiment and our initial work (Hagen et al., 2020) was reasonable (albeit unnecessarily complex).

Interestingly, Figure 8 reveals further interactions between the type of information encoded by different sets of sensory data and ANN structure. First, as mentioned above, some data sets are better than others. And second, increasing the number of nodes does not always improve performance. Case in point are the ANNs trained only with motor information. They always under-performed compared to ANNs trained with tendon tension, and had increasingly poor performance as the number of hidden-layer nodes increased.

### 3.3. Neural Network Performance Across Sensory Sets

Figures 7, 8 reveal that for ANNs with 15 hidden-layer nodes that were trained with 15 s of motor babbling performance (*i*) was consistent across sinusoidal and point-to-point movements for each sensory set (i.e., similarly small relative standard deviations) and (*ii*) only improved slightly when either babbling duration or hidden-layer nodes were increased for ANNs trained with tendon tension (*orange* and *dark blue* in both figures). For ANNs trained with tendon tension, the choice of 15 hidden-layer nodes and 15 s of babbling produced very accurate joint angle estimates with average errors ~10^−3^ degrees. This demonstrates a remarkable improvement in both accuracy, speed, practicality, and computational efficiency when compared to the ~10^0^ degree average errors obtained after 2 min of motor babbling reported in Hagen et al. (2020). Therefore the choice to use 15 s motor babbling duration and 15 hidden-layer nodes when performing the sensitivity analysis discussed in the following sections is justified.

**Figure 9:**
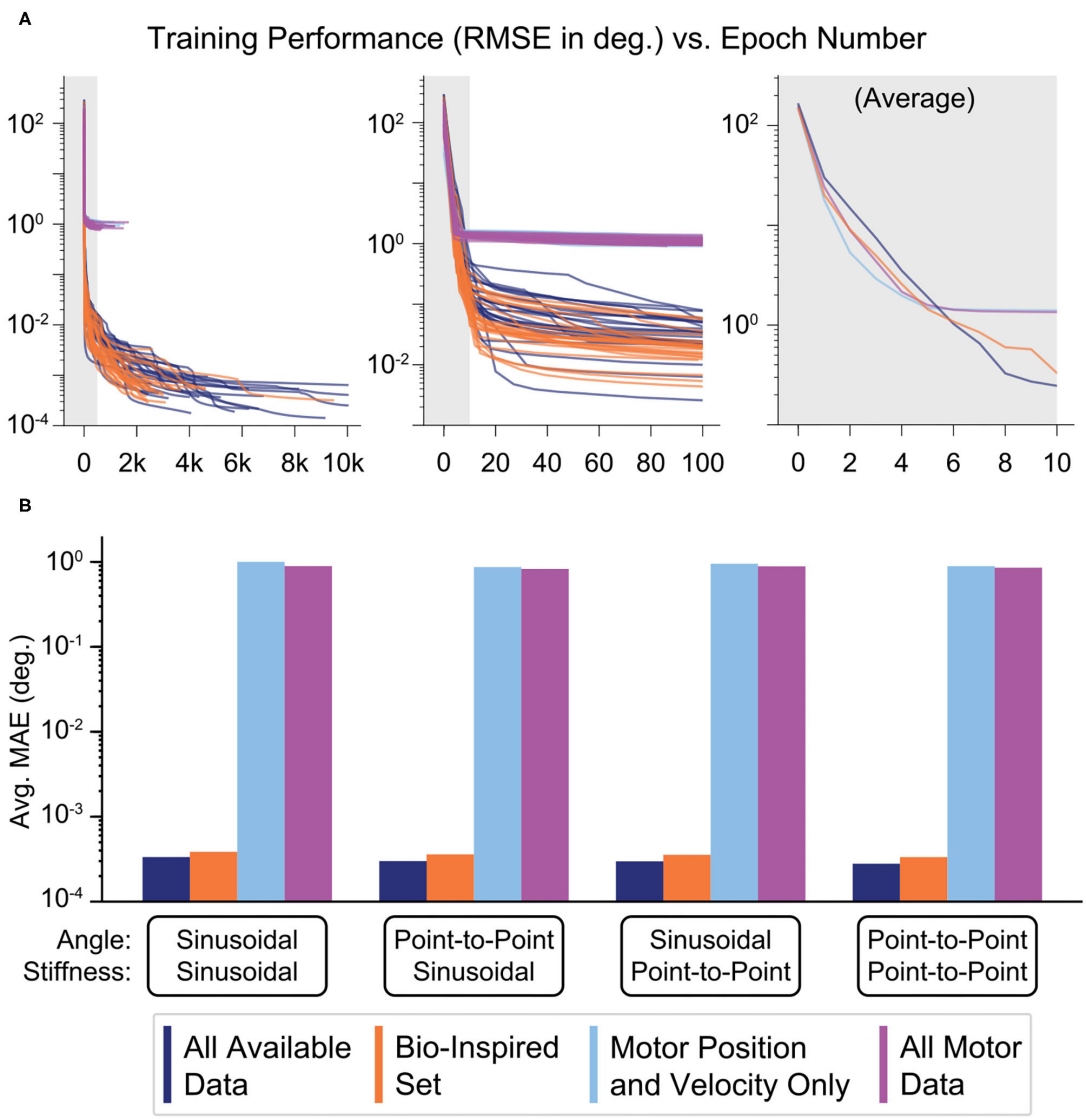
**(A)** Performance (RMSE in degrees) vs. the number of epochs needed to train each ANN. For each of the four sensory sets, 25 motor babbling simulation of 15 s were performed to train ANNs with 15 hidden-layer nodes. Although it took the ANNs more than 1,000 epochs for the performances to converge (even requiring up to 10,000 epochs for the ANNs trained with tendon tension data), the majority of the performance improvement came within the first 20–50 epochs (*middle*). In fact, the ANNs trained only with motor data (*Motor Position and Velocity Only* and *All Motor Data*) converged with as little as 6 epochs (as seen by the average plot on the *right*). Lastly, it appears that learning from motor information may have allowed for faster learning, but the performance was soon beaten by the ANNs trained with tendon tension (which took longer to learn). **(B)** Bar plots of the average performance (MAE) of each of the four ANNs when predicting joint angle from the four generalization movements (plotted on a log scale. For each sensory set, there is little difference across movements, but there is a consistent trend that the sensory sets that include tendon tension (*All Available Data* and the *Bio-Inspired Set*) perform 3 orders of magnitude better than the sets trained without tendon tension.

During training, the more accurate ANNs showed steep improvements followed by slow asymptotic progress, as seen by the plot of performance against the total number of epochs for each of the 25 ANNs trained for each sensory set (Figure 9A, left). The more accurate ANNs using the *Bio-Inspired Set* and the set of *All Available Data* utilized 2,917 and 5,284 epochs on average, respectively. In contrast, ANNs not using tendon tension converged well before 1,000 epochs. However, the middle panel in Figure 9A shows that the performance of the ANNs trained with tendon tension already performed two orders of magnitude better than their tension-less counterparts after 100 epochs, as they progressed in their steep improvement. In fact, if we average the training performance for the first ten epochs for each sensory set (Figure 9A, right) we found that (*i*) ANNs trained with motor information alone saturated their improvement around the 6th epoch and (*ii*) performed marginally better than ANNs trained with tendon tension before the 6th epoch. This implies that it is faster to learn a kinematic relationship between motor position and velocity information *but* the relationship is fundamentally of limited utility. Conversely, it took longer to learn an accurate mapping of the more complex kinematic *plus* kinetic relationship between motors, tendon tensions, and the joints that they actuate but the performance was drastically better. When the ANNs are allowed to converge without an epoch limit (i.e., as in Figure 9A, *left*), we again see that, across the four generalization tasks, the *average* performance of ANNs trained with tendon tension data is 2-3 orders of magnitude better than that of ANNs trained without it (Figure 9B).

### 3.4. Neural Network Performance Across Types of Movements

**Figure 10:**
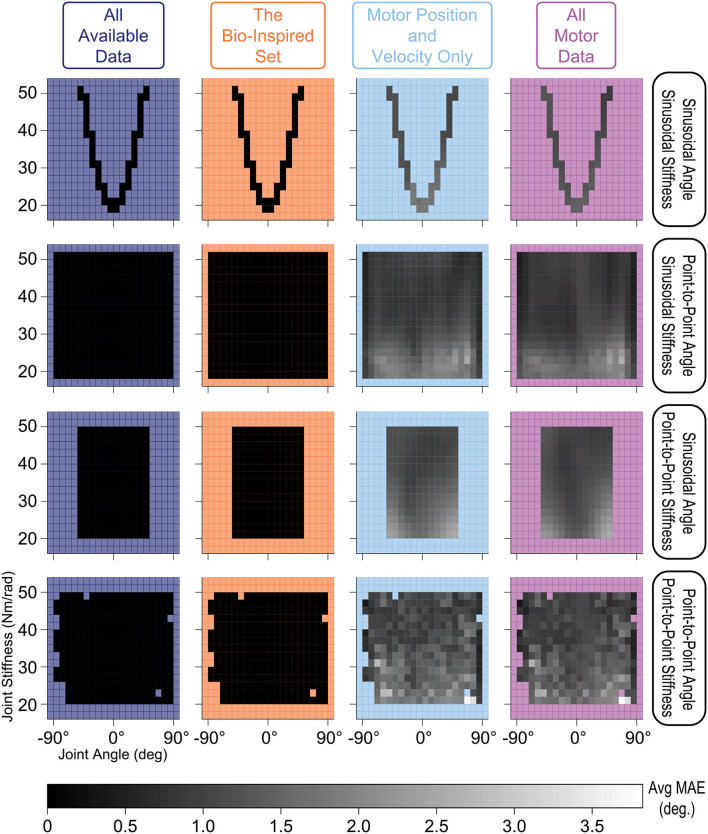
Linear-scale heatmap of the average MAE vs. joint angle and joint stiffness for sinusoidal and point-to-point trajectories (default *Low* tendon stiffness experiment). It is clear that the ANNs trained with tendon tension (*left two columns*) reliably predicted joint angles at any level of joint stiffness, while the ANNs trained without tendon tension (*right two columns*) had difficulty at low joint stiffness values (regardless of the type of movement). This is because tendon tensions that produce lower joint stiffness occupy the more nonlinear “toe” region of the tension-stretch relationship (i.e., more disproportionate lengthening per unit force) which causes more nonlinear decoupling between motor and kinematics states.

We also explored how the *type of time-varying sensory data* affected the estimates of joint angles. We compared performance across joint angle and joint stiffness values for each of the four movements and each sensory set by evenly dividing the output space into bins (every 9° and 2 Nm/rad) and calculating their average performance (Figure 10). ANNs trained with tendon tension performed equally well for all values of joint angle and joint stiffness across all movements (*dark shading—linear scale—of the stiffness vs. joint angle planes in the left two columns*). Alternatively, ANNs trained *without* tendon tension (*right two columns*) performed worse at *low* values of joint stiffness across all movements. These results make logical sense as lower *joint* stiffness can be attributed to the tendons operating in regions of lower *tendon* stiffness (called the “toe region” for physiological tendon) which is categorized by more disproportionate lengthening per unit force and more *nonlinear* decoupling between motor and kinematics states.

### 3.5. Sweeping Across Movement Frequencies

**Figure 11:**
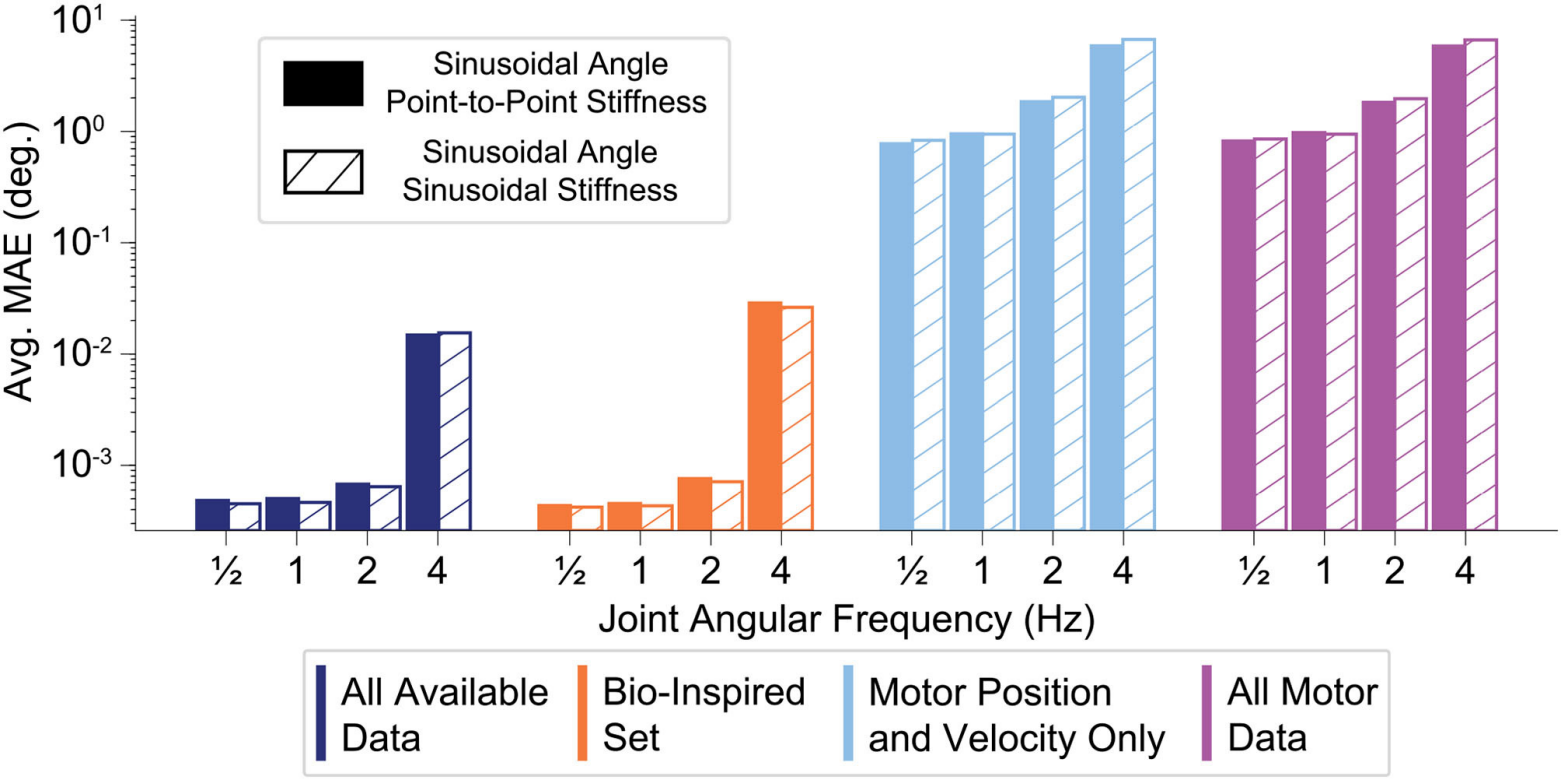
Bar plot of the average performance (MAE) of each sensory set (N=50 ANNs) as function of the frequency of the sinusoidal joint angle trajectory. The joint stiffness was either varied sinusoidally or by a point-to-point task (18)–(19). The ANNs trained with tendon tension (*left two sets*) appear to generalize better to higher frequency movements, only worsening slightly when the movements becomes fastest. The ANNs trained only with motor information may decrease their performance by a similar order of magnitude, but there is quite a difference between producing average errors of 10^−2^ and 10^1^ degrees.

The performance of all sensory sets decreased as the movement frequency increased (for either sinusoidal or point-to-point joint stiffness task), but the ANNs trained with tendon tension generalized better to faster movements (Figure 11). Note that Figure 11 is plotted on the log scale. While the *relative* decrease in performance for ANNs trained with tendon tension is larger at higher frequencies, the *absolute* decrease in performance can be considered small ( 10^−3^– 10^−2^ degrees) compared to the ANNs trained without tendon tension ( 10^0^ to 10^1^ degrees).

**Figure 12:**
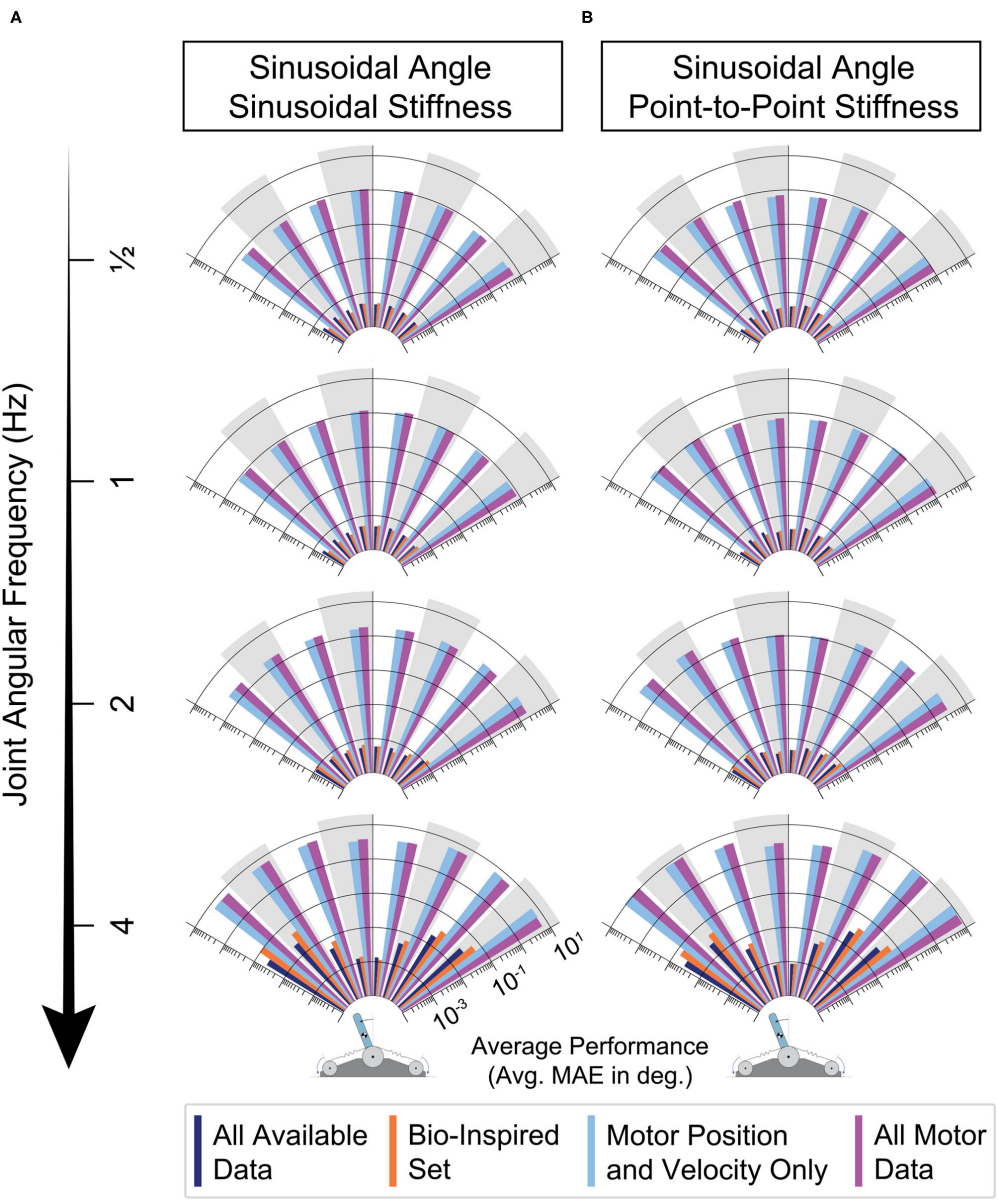
Radial bar plot of the average performance (MAE) of each sensory set across the joint angle space as the joint angular frequency is increased and the joint stiffness was either varied **(A)** sinusoidally (twice the frequency) or **(B)** as a point-to-point task. For either task, ANNs trained with tendon tension (*dark blue* & *orange*) generalized better to higher frequency movements, only worsening slightly when the movements becomes fastest. For these two sets, when movements were the fastest, the largest errors occurred at the boundaries of the sinusoidal movement (à* la* speed-accuracy trade offs).

By dividing each movement into joint angle bins (every 15 degrees) and calculating the average performance of each bin of all 50 ANNs trained for each sensory set, we can identify if errors in joint angle estimation have any dependence on the location in the work space as frequency is increased. The results *across frequencies* are plotted for each sensory set as logarithmic radial bar plots in Figure 12 for all 8 movement tasks. First, it is important to note that the ANNs trained only with motor information (*light blue* and *purple*) are relatively consistent across the joint angle space regardless of the choice of joint stiffness task—the performance of these ANNs was consistently poor everywhere for each frequency. Secondly, and perhaps more interestingly, for either joint stiffness task the performance for ANNs trained with tendon tensions (*dark blue* and *orange*) was consistent across the workspace for lower frequency movements, but developed edge effects as the movement became faster (i.e., when the movement reversed). This apparent *speed-accuracy* trade off has important consequences to the observed physiological phenomenon of the same name as mentioned in the Discussion.

### 3.6. Sweeping Plant Parameters

**Figure 13:**
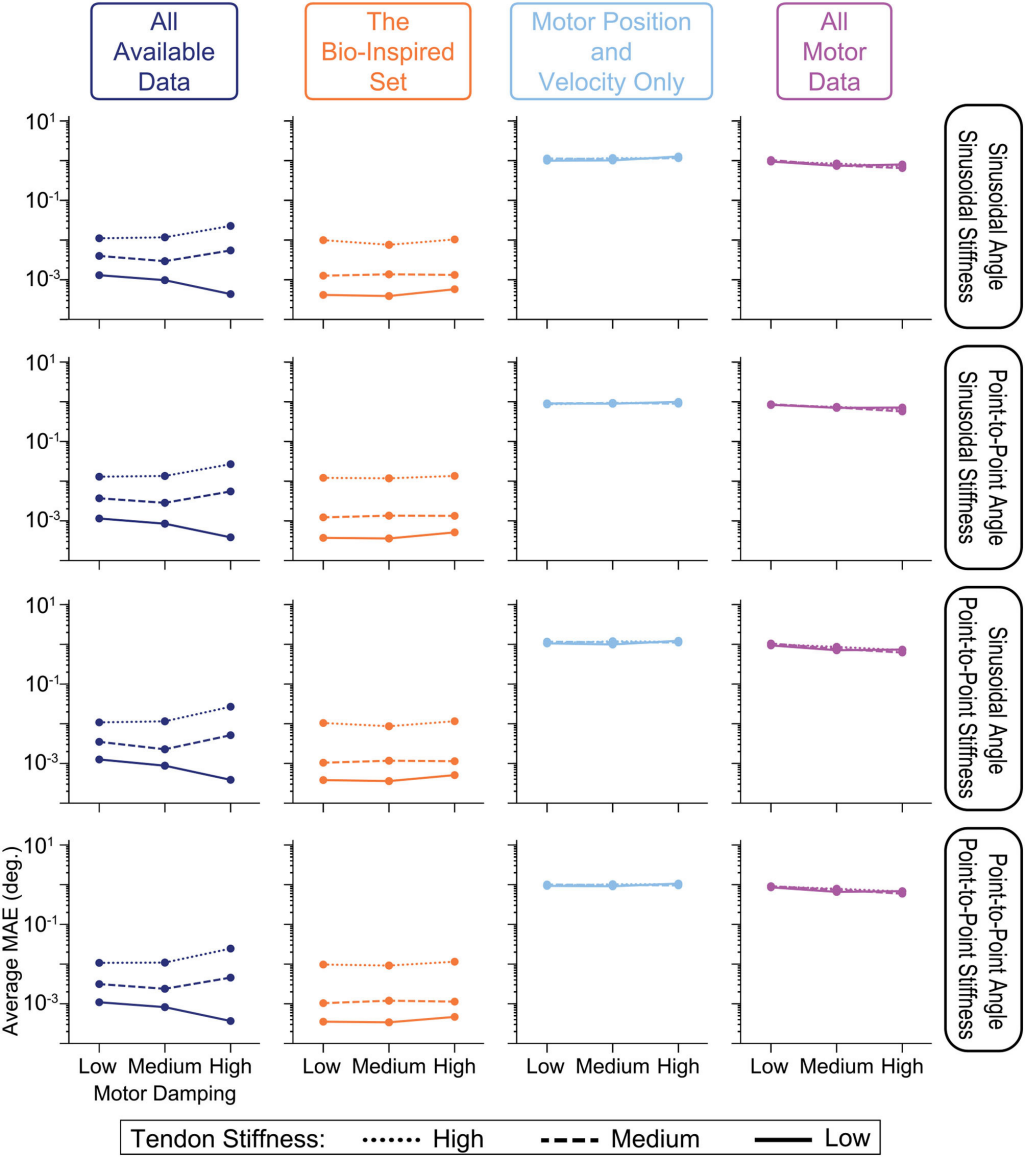
Comparing the average performance of ANNs designed to predict joint angles from one of four sensory sets when motor damping (*x-axis*) and tendon stiffness (*line style*) are varied. We see from the vertical separation of the lines in the left two columns that ANNs trained with tendon tension information perform worse as tendons become more inextensible—a trend not observed in the ANNs trained with motor information only (*right two columns*). Additionally, there appears to be no trends in performance with respect to motor damping *except* for those ANNs trained with all motor data (including acceleration, *right column*) or the set of *All Available Data* when tendon stiffness is *Low*, which intuitively makes sense as higher damping may mean more useful information in the motor acceleration data.

The performance of each sensory set across across all 4 generalization movements are plotted for each of the 9 combinations of motor damping and tendon stiffness parameters in Figure 13. It can be seen that the performance of ANNs trained with tendon tension information actually decreased as the tendons became stiffer (as indicated by the separation of lines in the left two columns of Figure 13), but still clearly outperformed the ANNs trained only with motor information by roughly 2 orders of magnitude. There do not appear to be any clear trends, however, when comparing performance and the amount of motor damping used. There is a slight positive trend (negative slope) between the performance of the ANNs trained with the *All Motor Data* and the amount of motor damping, as can be seen in the last column of Figure 13, but it is not significant. Similarly, a slight trend exists for ANNs trained with the set of *All Available Data* when the tendons are compliant (i.e., *Low* stiffness) that disappears when the stiffness increases. Interestingly (but not surprisingly), these two sensory sets are the only ones that include motor acceleration, and it would be expected that higher motor damping would cause motor acceleration data to become more useful leading to better performance in the ANNs that utilize it.

### 3.7. Very High Tendon Stiffness Experiment

**Figure 14:**
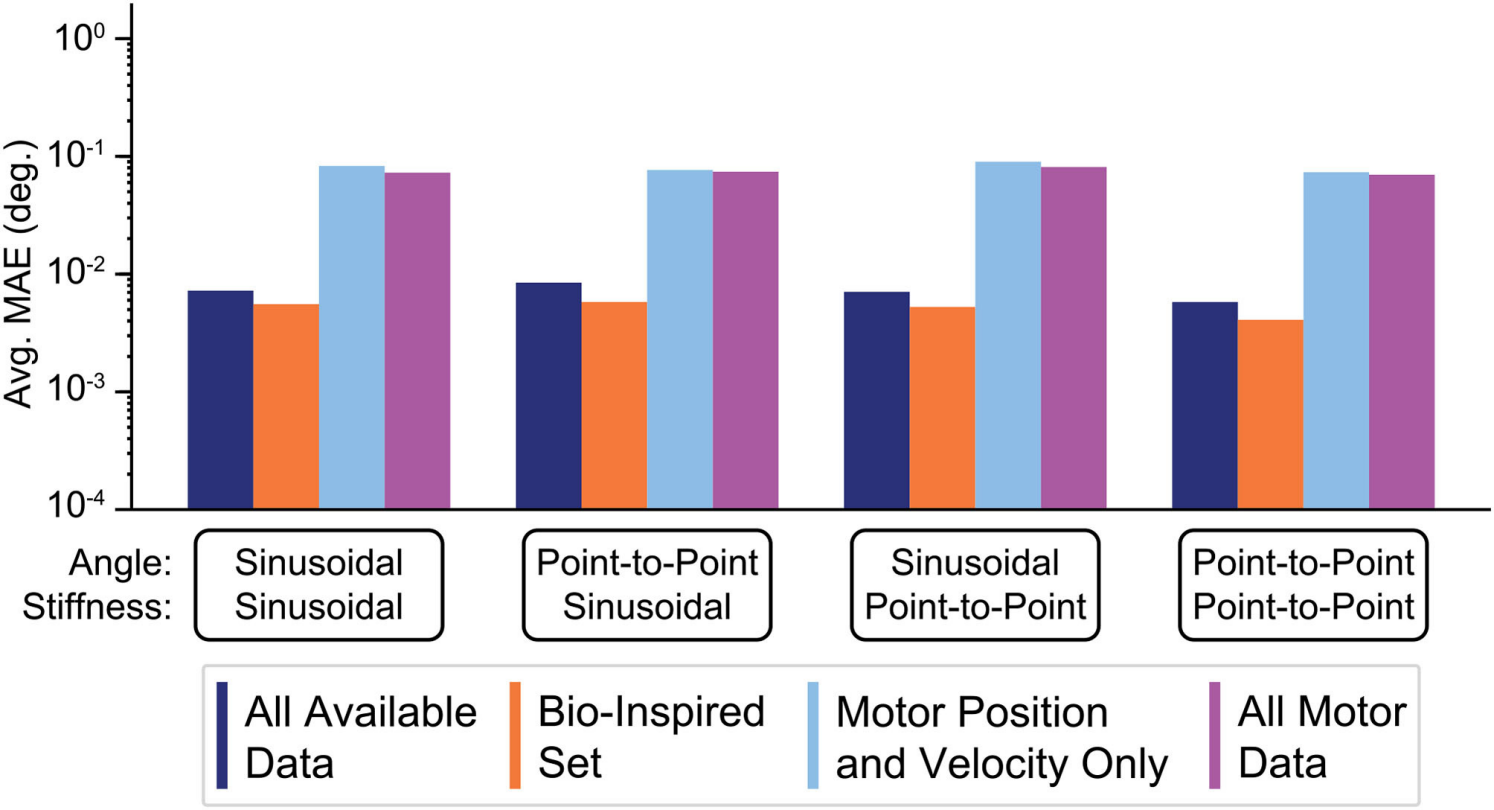
Bar plots of the average performance for ANNs for each sensory set when the tendon stiffness values are *very high* (see section 2.9 for explanation). ANNs trained with tendon tension information (the *Bio-Inspired Set* and the set of *All Available Data*), still outperform those ANNs trained with motor information alone (the sets of *Motor Position and Velocity Only* and *All Motor Data*). However, the difference in performance was reduced from 3 orders of magnitude (observed previously) to 1 because (*i*) the ANNs trained only with motor information improved their performance by nearly 1 order of magnitude and (*ii*) the ANNs that train with tendon tension information continue the trend of worsening performance when tendon stiffness increases (increasing errors by nearly 1 order of magnitude).

This last experiment tested the extreme case of using *very stiff tendons* as engineers tend to prefer designing robots with stiff tendons to better justify neglecting (potentially nonlinear) tendon stretch. The average performances of ANNs *(N = 50)* trained with these four sensory sets when asked to generalize to these movement tasks with higher joint stiffness values are shown Figure 14. Consistent with the results above, ANNs trained with tendon tension information still outperformed those ANNs trained without it, but the difference was reduced from 3 orders of magnitude to 1. That is, tendon tension information became less useful as the tendons approach inextensibility, *but not useless!*). Additionally, ANNs trained without tendon tension increased their performance from ~10^0^ to ~10^−1^ (cf. Figures 13, 14, respectively), consistent with our intuition that ANNs trained on systems with more inextensible tendons should see an increase in their performance because the behavior of the motors and the joint will be more coupled.

**Figure 15:**
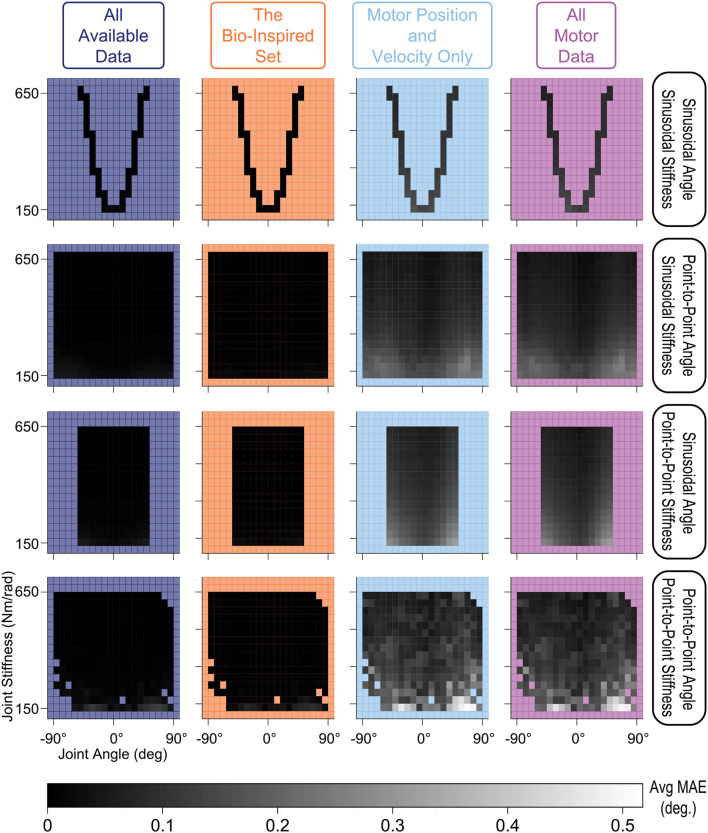
Linear-scale heatmap of the average MAE vs. joint angle and joint stiffness for sinusoidal and point-to-point trajectories (*Very High* tendon stiffness experiment). Similar to the default *Low* tendon stiffness results plotted in Figure 10, the ANNs trained with tendon tension data (*left two columns*) still (*i*) perform better and (*ii*) better generalize at lower stiffness values than ANNs trained only with motor data (although the difference is not as large).

For this very high stiffness case, we divided the joint angle and joint stiffness space into bins (every 9° and 32 Nm/rad.) and calculated their average performance, displayed as heat maps in Figure 15, to identify any trends across the output space for each sensory set and movement. Similar to the default *Low* tendon stiffness results shown in Figure 10, the ANNs trained with tendon tension data (*left two columns*) still (*i*) performed better across all movements and (*ii*) generalized better to lower stiffness values than ANNs trained only with motor data (*right two columns*). While the difference between ANNs trained with and without tendon tension is no longer as pronounced, the overall trends were still the same: tendon tension information was always useful—especially at low joint stiffness values.

## 4. Discussion

By expanding on our prior work, we now demonstrate a robust framework that can estimate joint angles in tendon-driven systems on the basis of sets of sensory data extracted from limited motor babbling. Importantly, the *Bio-Inspired Set* of sensory data performed best. This set mirrors the information available from muscles, which vertebrate animals use apparently seamlessly in order to estimate limb posture from sensors that are not located at joints (e.g., muscle fascicle length via muscle spindles and tendon tension via Golgi tendon organs). We find that for different durations of motor babbling (i.e., amounts of data) and different ANN architectures (i.e, network structures and number of nodes), the ANNs trained with data sets that include tendon tension outperformed the ANNs trained without it (Figures 7, 8). Intriguingly, those ANNs trained with the *Bio-Inspired Set* performed as well (if not better than) the baseline set of *all* motor and tendon tension sensory data, (*All Available Data*). Therefore, we conclude that it is possible to train an ANN on limited non-collocated measurements of *motor position, motor velocity, and tendon tension only* to estimate joint angles reliably during a variety of joint angle and joint stiffness trajectories. While the 2-tendon 1-joint architecture (2N design) discussed here is the most popular in tendon-driven robots (with each pair of tendons acting only on a single joint), these results can be readily extended to robots with multiple joints.

### 4.1. Robustness Over the Parameter Space

Our findings are important to autonomous learning, control, and on-line adaptation in physical robots because only 15 s of motor babbling sufficed to produce consistent results for each of the four sensory sets when used to train ANNs with 15 hidden-layer nodes. This may be a consequence of the babbling patterns chosen, which we designed to broadly span the joint angle and joint stiffness spaces with a high degree of temporal correlation, similar to that seen during biological babbling in infants (Dominici et al., 2011; Marjaninejad et al., 2019b). More specifically, we illustrated in Figures 7, 8 that increasing either the number of hidden-layer nodes or the duration of motor babbling only slightly improved the performance of ANNs trained with tendon tension allowing us to provide very accurate joint angle estimates with average errors ~10^−3^ degrees without having to expose the plant to large training times [compared to the ~10^0^ degree reported in Hagen et al. (2020)]. One may argue that such sub-degree improvements in estimation errors are not relevant. We argue, however, that they are important when controlling nonlinear systems like bio-inspired tendon-driven limbs. Improving the stability margin and robustness of optimal control methods for such systems has been a longstanding goal (Doyle, 1978; Weghe et al., 2004; Theodorou et al., 2011; Reed et al., 2020). Improving the joint angle estimates can only facilitate and improve their control. Small errors in the proximal joints of multiarticular limbs result in large errors of end-effector position, which explains the much larger number of muscle spindles in proximal vs. distal limb muscles (Scott and Loeb, 1994). Moreover, brief periods of retraining (using data coming from another sensory source such as visual feedback) may be particularly useful to compensate for errors caused by, say, sensor drift or plant wear. The cerebellum appears to perform such a role to fine-tune neural networks associated with gaze control (Koziol et al., 2014).

Alternatively, we found that ANNs that trained only with motor kinematic information performed their best when the networks had *one* hidden-layer node implying that the best approximation these ANNs trained *without* tendon tension could provide was captured by a single equation of motor positions and velocities. Adding nodes without providing any additional useful information resulted in worse performance as more floating parameters (i.e., weights and biases) needed to be set from limited data to recapture what was best described by a simple equation. This single-equation approximation is reminiscent of approximations of muscle lengths that rely on the “inextensible tendon” assumption used in many musculoskeletal models, which results in large errors in muscle fascicle length and velocity estimates, particularly in pinnate muscles (Hagen and Valero-Cuevas, 2020). Here we demonstrate the consequences of such errors for limb position estimation, and their mitigation by including tendon tension in the estimator.

When comparing the average *training* performance of these ANNs (Figure 9A, right) we found (*i*) that ANNs trained with motor information alone only marginally improved after the 6th epoch and (*ii*) that before the 6th epoch the performance of the ANNs trained with tendon tension information was *worse*. These observations imply that it is initially easier to learn the relationship between motor position and velocity information *but* the relationship is fundamentally incomplete and further training cannot rectify this. Conversely, it took longer to learn the complex relationship between motors, tendons, and the joints that they actuate, but the performance was drastically better. These results further strengthen the argument that tendon tension information critically enables the accurate prediction of joint angles from non-collocated sensory information.

Regarding speed of movement, we found that the ANNs that trained with tendon tension information in addition to motor kinematic information (e.g., the *Bio-Inspired Set* of sensory information) generalized better to faster movements than ANNs that trained without tendon tension data, producing average prediction errors ≤ 10^−2^ degrees. Higher frequencies of oscillatory movement necessarily require larger torques for acceleration and deceleration, which results in increased tendon stretch. Therefore, these results are consistent with our expectations that the performance of the ANNs trained only with motor information should generalize poorly to these more demanding, more rapid movements while ANNs that train with tendon tension information should generalize well as they can correct for large changes in tendon behavior responsible for decoupling motor and joint angle states. Interestingly, ANNs trained with tendon tension exhibited edge effects during high frequency movements, with lower performance at the points of movement reversal (Figure 12). As this effect was present for the baseline set of *All Available Data*, it is unlikely that this phenomenon can be attributed to missing or incomplete input data. Instead, because higher frequency movements will naturally require larger tendon tensions in order to brake the joint as it approaches the target amplitude (i.e., the point at which the joint reverses direction), the tendon tension data—which we have shown to be *critical* for accurate joint estimation—may move outside its dynamic range causing the ANNs to produce less accurate estimates as they try to *extrapolate* this relationship. Such a speed-accuracy computational trade-off has been well established in humans where faster movements come at the cost of lower accuracy at the movement target (Fitts, 1954). Here we present a complementary state-estimation mechanism for a similar trade-off.

### 4.2. Relationships Between Robotic and Biological Systems

For the extreme case of nearly-inextensible tendons, ANNs trained only with motor information improved substantially, although they were still about an order of magnitude poorer than ANNs trained with tendon tension (Figure 14). This confirms the intuition of robot builders who have long used such stiff tendons despite their vulnerability to breakage or risk of injuring human users. Assuming inextensible tendons carries well-known risks to state estimation and controller robustness. To our knowledge, we are the first to quantify those risks and, more importantly, confirm that they can be mitigated by incorporating sensors of tendon tension. Such a biological strategy has been proposed (Scott and Loeb, 1994). The ~100 Golgi tendon organs distributed throughout the myotendinous junction of the typical muscle appear to be well-suited to generating an ensemble signal that accurately, albeit nonlinearly, reflects total tendon tension (Mileusnic and Loeb, 2006). The requisite integration of signals from tension-sensing Golgi tendon organs and length-sensing muscle spindles has been identified in the spinocerebellar tracts (Bosco and Poppele, 1997, 2001). The work presented here demonstrates that a neural network can be trained to use such proprioceptive information to compute accurate postures, as suggested previously (Scott and Loeb, 1994; Dimitriou and Edin, 2008; Van Soest and Rozendaal, 2008; Kistemaker et al., 2013). The choice of a nonlinear spring for our tendon model was based on biological tendons and aponeuroses (Scott and Loeb, 1995). The lower-stiffness “toe” region at low tension is associated with disproportionate tendon stretch per unit tendon tension and, therefore, larger and more nonlinear decoupling between motors and joint angles (Zajac, 1989; Hagen and Valero-Cuevas, 2020). Networks trained with tendon tension data did not have problems generalizing to these lower joint stiffness (and therefore lower tendon stiffness) tasks, suggesting that tendon tension data were sufficient for ANNs to model this nonlinearity accurately.

Mindful of the robotic vs. biological distinction, we have been careful not to call joint angle the “state” and we do not call our approach “state estimation.” It is not clear what “state” means in the biological context. Rather we used the ANN approach as a generic means to answer the information-theoretical question of whether and how different sets of afferent (sensor) information can—in principle—estimate a variable of interest: joint angles in this case.

In engineering, in contrast, the concept of state is well-defined and central to the Newtonian, Lagrangian, Kanesian, and Hamiltonian approaches to rigid body dynamics and their control: the “states of a system” are the minimal set of generalized coordinates (usually kinematic DOFs in robotic systems) that suffice to explain the energy transformations the system can undergo, and how to control them. In this context, the concepts of state, observability, and the estimation of state as a function of sensory information (i.e., state estimation) are clearly defined. Biological controllers are hierarchically organized into computational subsystems that may employ different coordinate frames (Soechting and Flanders, 1992; Scott and Loeb, 1994), none of which may correspond to a canonical physical descriptor such as joint angles. Limb posture must be derived from sensors in complex arrays of muscles and tendons that often cross multiple joints, each with more than one degree of freedom (Scott and Loeb, 1994).

## 5. Conclusion

We have demonstrated that it is possible to utilize sensors not located at joints in tendon-driven systems to provide accurate joint angle estimation during dynamical tasks using neural networks trained with limited data—a novel bio-inspired posture estimation framework called *insideOut*. Specifically, we have shown that tendon tension data in addition to motor position and velocity data were sufficient to train ANNs capable of accurately predicting joint angles in compliant tendon-driven systems with as little as 15 s of motor babbling data. More importantly, these joint angle estimates were robust to changes to the physical characteristics of the system (e.g., tendon stiffness and motor damping) and tasks (e.g., joint angle and joint stiffness trajectories). Our findings have important implications to autonomous learning, control, and on-line adaptation in tendon-driven robots as we present an efficient data-driven approach that creates an implicit model that accurately maps limited sensory information to limb posture (i.e., joint angle). Future work will deploy this algorithm in physical robots with multiple joints to demonstrate its practical utility and to explore the effect of more complicated routings for tendons that span more than one joint, as is common in biological musculoskeletal systems.

## Data Availability Statement

The datasets presented in this study can be found in online repositories. The names of the repository/repositories and accession number(s) can be found below: https://github.com/danhagen/insideOut.

## Author Contributions

In particular, DH, AM, GL, and FV-C contributed to the design of this work. DH and AM laid the foundation for the ANN framework. DH, AM, and FV-C participated in the writing of this manuscript. GL provided valuable research guidance and edited the manuscript. All authors listed have made a substantial, direct and intellectual contribution to the work, and approved it for publication.

## Funding

This research was supported by the National Institute of Arthritis and Musculoskeletal and Skin Diseases of the National Institutes of Health under award numbers R01 AR-050520 and R01 AR-052345, National Institute of Neurological Disorders and Stroke of the National Institutes of Health under the award number R21-NS113613, Department of Defense CDMRP Grant MR150091, and Awards W911NF1820264 and W911NF2120070 from the DARPA-L2M program to FV-C. We acknowledge additional support from the Graduate School of the University of Southern California in the forms of Provost (DH and AM) and Research Enhancement (AM) Fellowships.

## Author Disclaimer

The content of this endeavor is solely the responsibility of the authors and does not represent the official views of the National Institutes of Health or the Department of Defense.

## Conflict of Interest

The authors declare that the research was conducted in the absence of any commercial or financial relationships that could be construed as a potential conflict of interest.

## Publisher's Note

All claims expressed in this article are solely those of the authors and do not necessarily represent those of their affiliated organizations, or those of the publisher, the editors and the reviewers. Any product that may be evaluated in this article, or claim that may be made by its manufacturer, is not guaranteed or endorsed by the publisher.

## Footnotes

^1^There are additional biological sensors that detect stretch in the skin and synovial capsule, but these do not *directly* encode joint position either (Kandel et al., 2000).

^2^Torque due to gravity: *G*(θ_*j*_) = −*m*_*j*_*gL*_*CM*_sin(θ_*j*_).

^3^The values of the parameters used in (1)–(2) were taken from Palli et al. (2008) & Hagen et al. (2020).

^4^These functions would require our numerical integration simulation to use a variable time step and the resulting dynamics complicate the control law for the feedback linearization technique used to produce the generalization trajectories.

^5^As described in Zajac (1989), the most important determinant of muscle-tendon interaction in biological cases is the ratio between tendon slack length (i.e., the length under zero tension) and the optimal muscle fascicle length (i.e., the length at which the maximum force can be generated), which is omitted here for simplification. In this idealized robotic analog system, motors can produce the same torque at any rotor angle. Therefore, a more appropriate metric would be the ratio between tendon slack length and the rotor's *moment arm* as this describes an actuator's excursion per unit motor rotation. For the assumed motor moment arm value (0.02 m) and tendon slack length range (0.01-0.04 m) this ratio is between 0.5 and 4, corresponding to the lower half of the reported physiological range for the ratio between tendon slack length and optimal muscle length ≤ 11.25; Zajac, 1989; Hoy et al., 1990). Future work will explore the affect of changes to this displacement-stretch ratio, but previous work and intuition suggest that increasing this ratio will increase the relative amount a tendon stretches compared to the amount by which a rotor displaces, causing more decoupling between motor data and joint angles and increasing the usefulness of knowing tendon tension data in the proposed ANN algorithm (Hagen and Valero-Cuevas, 2020).

^6^If there are asymmetries in either the actuators or the plant or if the system becomes more complicated with higher degrees of freedom or more motors, then this approach must be modified to consider the ratios between moment arms and the nullspace of joint torques as dictated by the limb's equations of motion (Valero-Cuevas, 2016).

^7^https://www.mathworks.com/help/deeplearning/ref/feedforwardnet.html.

^8^The Nguyen-Widrow algorithm is designed to reduce training time by distributing the initial weights and biases in such a way that the active region of each neuron in the hidden-layer is evenly distributed across the input space. As the goal of this experiment was to compare how well and how quickly ANNs trained with different sensory sets learned to predict joint angles, the Nguyen-Widrow algorithm was a poor candidate for initialization function as it may influence the average performance differently across ANNs trained with the four sensory sets.

^9^Note that Figure 7 is plotted on a log scale, the peaks for both the *All Available Data* and *Bio-Inspired Set* at 20 s of babbling are on the order of 10^−3^ to 10^−2^, and therefore do not reflect large variations from the average values but more likely noise.
